# Inhibition of (dppf)nickel-catalysed Suzuki–Miyaura cross-coupling reactions by α-halo-N-heterocycles†

**DOI:** 10.1039/d1sc04582b

**Published:** 2021-10-11

**Authors:** Alasdair K. Cooper, Megan E. Greaves, William Donohoe, Paul M. Burton, Thomas O. Ronson, Alan R. Kennedy, David J. Nelson

**Affiliations:** WestCHEM Department of Pure and Applied Chemistry, University of Strathclyde 295 Cathedral Street Glasgow G1 1XL Scotland UK david.nelson@strath.ac.uk; Chemical Development, Pharmaceutical Technology and Development, Operations, AstraZeneca Macclesfield SK10 2NA UK; Syngenta, Jealott′s Hill International Research Centre Bracknell Berkshire RG426EY UK

## Abstract

A nickel/dppf catalyst system was found to successfully achieve the Suzuki–Miyaura cross-coupling reactions of 3- and 4-chloropyridine and of 6-chloroquinoline but not of 2-chloropyridine or of other α-halo-N-heterocycles. Further investigations revealed that chloropyridines undergo rapid oxidative addition to [Ni(COD)(dppf)] but that α-halo-N-heterocycles lead to the formation of stable dimeric nickel species that are catalytically inactive in Suzuki–Miyaura cross-coupling reactions. However, the corresponding Kumada–Tamao–Corriu reactions all proceed readily, which is attributed to more rapid transmetalation of Grignard reagents.

## Abstract 2

Nickel complexes with a dppf ligand can form inactive dinickel(ii) complexes during Suzuki–Miyaura cross-coupling reactions. However, these complexes can react with Grignard reagents in Kumada–Tamao–Corriu cross-coupling reactions.

## Graphical abstract

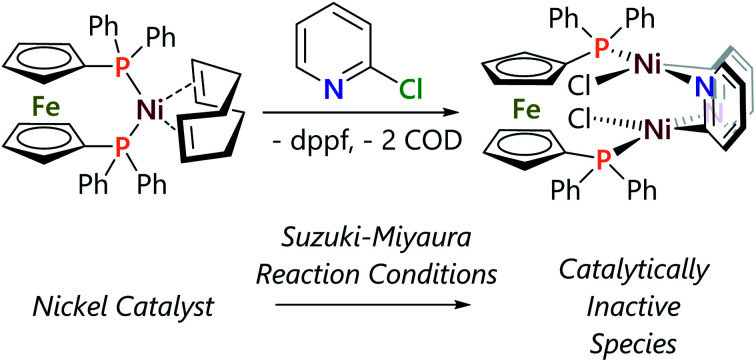

## Introduction

Palladium-catalysed cross-coupling reactions are arguably amongst the most important and impactful developments in organic synthesis in the past few decades. This was recognised by the award of the 2010 Nobel Prize in chemistry to Heck, Negishi, and Suzuki.^1,2^ Many of these reactions can also be catalysed by nickel,^3^ and as such the development and application of nickel catalysis in organic synthesis is an area of active development;^4^ research in this field has gone far beyond simply developing a cheaper alternative to palladium,^3^ and instead has allowed new mechanistic manifolds to be identified and exploited.^5,6^ Further developments in this field will inevitably rely on further developments in our understanding of the underlying reaction mechanisms and the effects of substrate structure on reactivity.

**Scheme 1:**
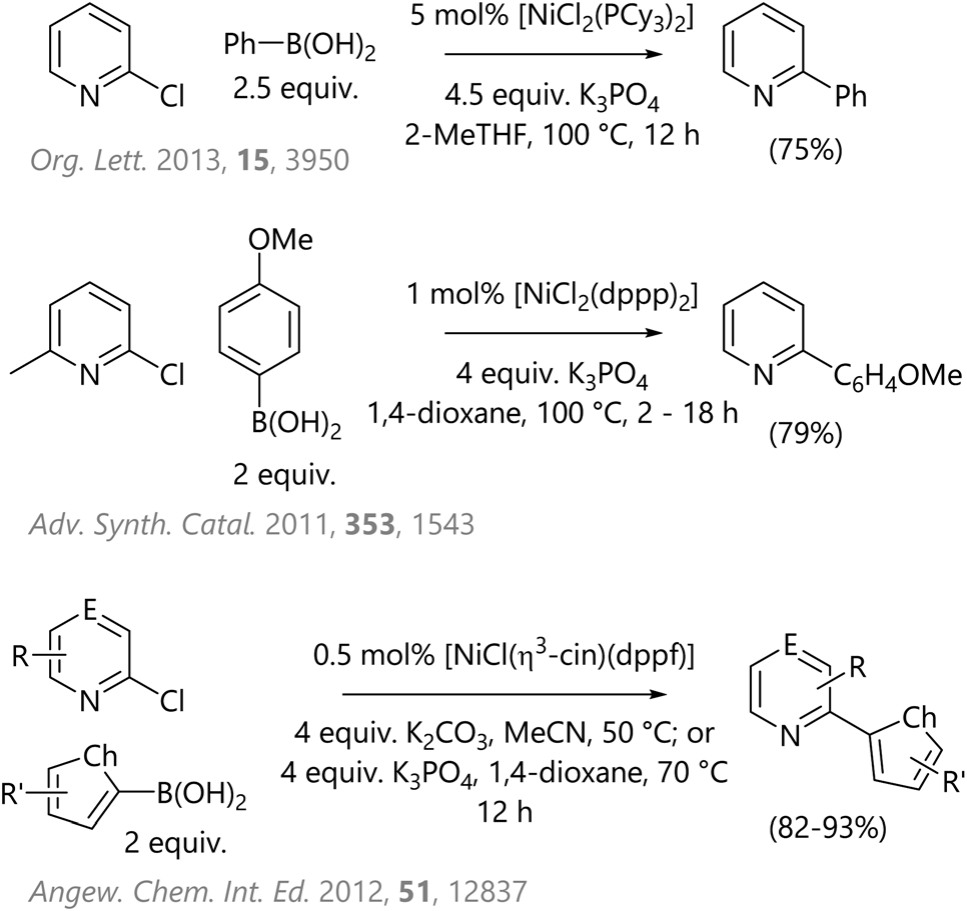
Literature examples of Suzuki–Miyaura cross-coupling reactions of 2-chloropyridine substrates (E = CH or N; Ch = O or S).

Heteroarenes are valuable motifs in a range of target molecules of interest to the pharmaceutical and agrochemicals industries, amongst others.^7,8^ Despite this, many synthetic methods that are readily deployed using aryl substrates are not easily applied to the synthesis of heteroaryl compounds. A salient example from cross-coupling chemistry is the poor stability of 2-pyridylboronic acids, such that ‘slow release’ alternatives such as trifluoroborates or MIDA boronates are required in order to achieve successful Suzuki–Miyaura cross-coupling reactions with high yields.^9^ Several studies have identified catalytic systems based on nickel that are competent for the Suzuki–Miyaura cross-coupling reactions of heteroaryl halides (Scheme 1).^10–12^ However, in two of these studies there is a single example of a 2-chloropyridine substrate; the third study contains a number of examples where 2-chloropyridine substrates undergo cross-coupling with 2-furanyl- and 2-thiophenylboronic acids.

Our research programme in the field of nickel catalysis has focussed on developing an understanding of reaction mechanisms and substrate/reactivity relationships.^13^ We have studied the reactions of aryl^14^ and alkyl halides^15^ with a prototypical dppf-nickel(0) complex, and established that aryl halide substrates that bear functionality such as aldehydes and ketones exhibit interesting and potentially exploitable behaviour in catalytic reactions.^16,17^ We were interested in how the fundamental reactivity of haloheteroarenes with nickel(0) differed from the reactivity of haloarenes, and how this might affect the outcomes of relevant cross-coupling reactions.

Here we describe the identification of dinickel(ii) species that readily form in catalytic reactions where α-halo-N-heterocycles are present, and where [NiCl(*o*-tol)(L)] complexes^18^ are used as pre-catalysts; these dinickel(ii) complexes are inert towards transmetalation with organoboron and organotin reagents, but undergo reaction with Grignard reagents. These complexes are therefore not competent for Suzuki–Miyaura or Stille cross-coupling reactions but do not impede Kumada–Tamao–Corriu cross-coupling reactions. Given the importance of heterocyclic substrates in the synthesis of bioactive molecules, and the widespread utility of cross-coupling reactions in academia and industry,^19^ this represents a significant finding that will affect how synthetic chemists should apply some nickel-catalysed reactions in the course of their research programmes.

## Results and discussion

**Scheme 2:**
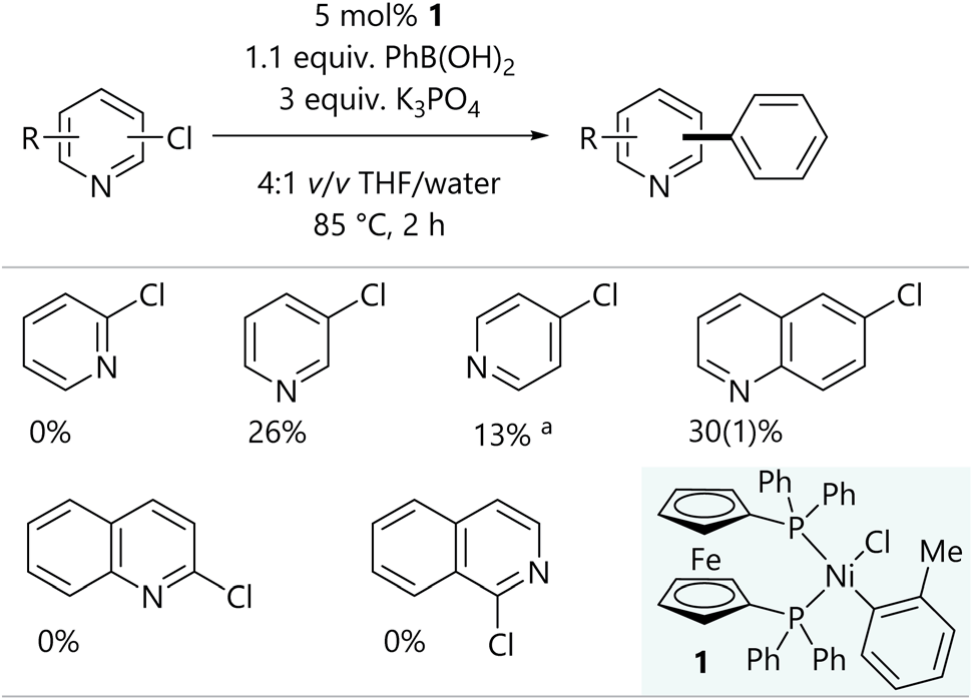
Initial attempts at the Suzuki–Miyaura reaction of chloroheteroarenes. Results are quoted as conversion to product as determined by calibrated GC-FID analysis. ^a^Substrate was used as the hydrochloride salt, so 4 equiv. K_3_PO_4_ were used.

Initial Suzuki–Miyaura cross-coupling reactions of 2-, 3-, and 4-chloropyridine were conducted using conditions that were previously used for aryl halides (Scheme 2);^16,17^ the readily-prepared bench-stable [NiCl(*o*-tol)(dppf)] pre-catalyst (**1**) was used.^18^ The conversions obtained for 3- and 4-chloropyridine were promising, but the lack of reactivity of 2-chloropyridine was rather unexpected. Further experiments established the reactivity of 6-chloroquinoline, but 2-chloroquinoline and 1-chloroisoquinoline led to no cross-coupled product.

**Scheme 3:**
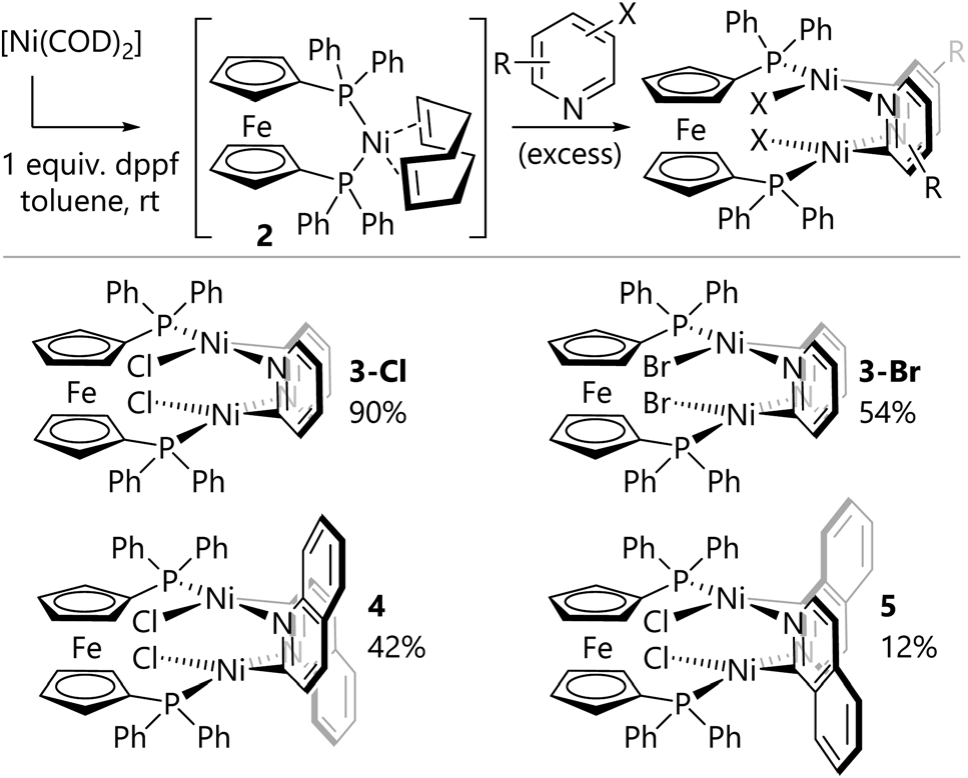
Synthesis of dimeric nickel(ii) complexes.

In order to gain some insight into the speciation of the catalyst during the reaction, an aliquot of the nickel-catalysed reaction of 2-chloropyridine was analysed by ^31^P{^1^H} NMR spectroscopy. Signals were observed that corresponded to free dppf (*δ*_P_ = −17 ppm, singlet) and a new diamagnetic species (*δ*_P_ = 22 ppm, singlet). The identity of the latter species was unclear, so reactions were carried out in which [Ni(COD)_2_] and dppf were first mixed in solution to form [Ni(COD)(dppf)] (**2**)^20^*in situ* before the addition of excess 2-chloropyridine (Scheme 3). The resulting deep red precipitate was analysed by ^31^P NMR spectroscopy, which indicated the presence of the new species as the major component (*δ*_P_ = 22 ppm, singlet). Complex **3-Cl** was obtained by filtration and washing with diethyl ether.

**Fig. 1:**
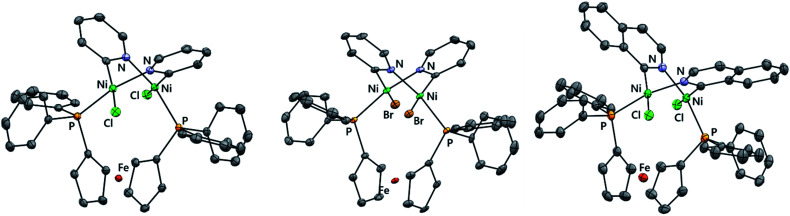
Molecular structures of **3-Cl** (left), **3-Br** (middle), and **5** (right) determined by single crystal X-ray diffraction analysis. Solvent molecules and hydrogen atoms are omitted for clarity.

Single crystals of [{NiCl(μ-2-py)}_2_(dppf)] (**3-Cl**) suitable for X-ray diffraction analysis were obtained serendipitously from an aged NMR sample and also from the careful crystallisation of isolated material (Fig. 1(a)). The crystal structure shows two nickel centres which are bridged by a single dppf ligand and by two 2-pyridyl units. Each nickel centre also bears a terminal chloride ligand.

The same synthetic route was deployed with different halogenated N-heterocycles to yield the corresponding complexes. Complexes **3-Br** (from 2-bromopyridine), **4** (from 2-chloroquinoline), and **5** (from 1-chloroisoquinoline) were obtained; complexes [{NiBr(μ- 2-py)}_2_(dppf)] (**3-Br**) and [{NiCl(μ-2-isoquin)}_2_(dppf)] (**5**) were characterised using single crystal X-ray diffraction analysis (Fig. 1(b) and (c)). The isolation of dinickel complexes in which the dppf ligand spans two nickel centres was unexpected, especially as dppf is typically considered to have a relatively narrow bite angle (*ca.* 99°).^21,22^ The cyclopentadiene ligands on iron in these three new complexes are significantly distorted from the parallel arrangement that is typically observed in mononuclear nickel complexes of the dppf ligand.

A search of the Cambridge Structural Database was conducted and a small number of analogous structures were identified with two nickel centres bridged by two 2-pyridyl units; their catalytic activity (or inactivity) has not been established. These feature monodentate ligands (PPh_3_ or PEt_3_) rather than bidentate ligands;^23–25^ these dimers are typically not cleaved upon the addition of excess triphenylphosphine or 1,2-bis(dicyclohexylphosphino)ethane, but can be broken up by the addition of triethylphosphine. An analogous complex has been reported with 1,3-bis(2,6-diisopropylphenyl)imidazol-2-ylidene (IPr) as the supporting ligand; [NiCl(μ-2-py)(IPr)]_2_) is catalytically competent for Buchwald–Hartwig amination reactions, but the corresponding trimeric structure is not.^26^

Palladium analogues of this structural motif, specifically [PdX(μ-2-py)(PPh_3_)]_2_ (X = Cl, Br), have been prepared and characterised.^27,28^ These palladium complexes are effective pre-catalysts for cross-coupling reactions, and were deployed as a source of mono-ligated “Pd(PPh_3_)”,^28^ whereas in the present study the dppf/nickel analogue hinders productive catalysis. It is unclear at this stage why this difference exists, but it might be that the smaller, harder nickel(ii) centre results in more favourable coordination of the nitrogen atoms in pyridine and related heteroarenes.

These results prompted further investigation of the oxidative addition of these heteroarenes to dppf-nickel(0); this investigation utilised the kinetic method that we have deployed previously, in which [Ni(COD)(dppf)] (**2**) is exposed to an excess (20 equiv.) of organohalide in benzene-d_6_ solvent (see the ESI†).^14^ The reactions were monitored using ^31^P{^1^H} NMR spectroscopy, and rate constants were obtained using a first order treatment of the decrease of the integral for **2** over time. The rate constants obtained are recorded in Table 1. Signals for **3-Cl** and **4** were observed in the ^31^P{^1^H} NMR spectra for the reactions of 2-chloropyridine and 2-chloroquinoline, respectively, consistent with their rapid formation immediately after oxidative addition, rather than their formation over the much longer timescales involved in the growth of single crystals for X-ray diffraction analysis.^29^ No corresponding signals were detected in the reactions of 3-chloropyridine and 6-chloroquinoline, and these most likely form [NiCl(dppf)] as the ultimate (paramagnetic) nickel-containing product.^14,17^

**Table 1:** Rate constants for the oxidative addition of haloheteroarenes (0.44 mol L^−1^) to [Ni(COD)(dppf)] (**2**) (0.022 mol L^−1^) in benzene-d_6_ solution

Entry	Substrate	*T* (K)	*k*_obs_ a (s^−1^)
1	Chlorobenzene	343	3.7(4) × 10^−3^
2^b^	Chlorobenzene	323	3.75(7) × 10^−4^
3	6-Chloroquinoline	323	2.51(1) × 10^−3^
4	Chlorobenzene	293	—c
5	2-Chloropyridine	293	3.5(2) × 10^−3^
6	3-Chloropyridine	293	2.7(1) × 10^−3^
7	1-Chloroisoquinoline	293	2.6(3) × 10^−3^
8	2-Chloropyridine	283	9.8(3) × 10^−4^
9	2-Chloroquinoline	283	2.62(9) × 10^−3^

a Average of two experiments.

b From ref. 14.

c No conversion after 8 h.

Palladium catalysts are known to undergo much faster oxidative addition to 2-halopyridines than the corresponding halobenzene and the corresponding oxidative addition reactions involving nickel show the same trend.^30^ These data show that the oxidative addition reactions of chloro-N-heterocycles are significantly faster than the oxidative addition of chlorobenzene. Attempts were made to gather kinetic data for the oxidative addition of chlorobenzene to **2** at 293 K, but the reaction was far too slow for practical *in situ* monitoring. The extension of the π-system to quinoline or isoquinoline further increases the reactivity relative to that of pyridine, consistent with the previously observed trend when moving from phenyl to naphthyl.^14^

**Scheme 4:**
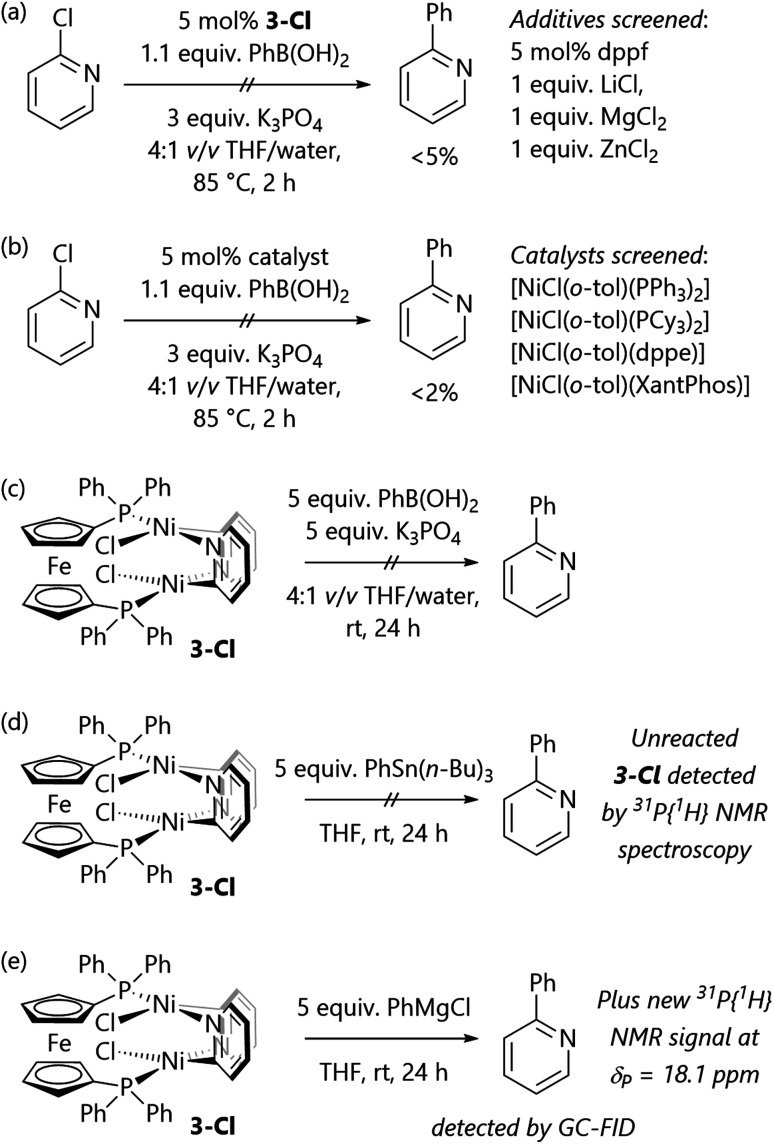
(a) Complex **3-Cl** is does not catalyse Suzuki–Miyaura cross-coupling reactions. (b) Alternative catalysts do not catalyse these reactions. (c) Complex **3-Cl** does not react with base and boronic acid. (d) Complex **3-Cl** does not react with PhSn(*n*-Bu)_3_. (e) Complex **3-Cl** reacts with PhMgCl.

These results all suggest that the poor reactivity of **3-Cl** is the underlying cause of the failure of the Suzuki–Miyaura cross-coupling reactions of α-chloro-N-heterocycles. Complex **3-Cl** is not a competent pre-catalyst for Suzuki–Miyaura reactions, even in the presence of additional dppf (Scheme 4(a)); attempts to use Lewis acids (LiCl, MgCl_2_, ZnCl_2_) to shift the equilibrium towards monomeric species and enable cross-coupling chemistry were unsuccessful.

A set of pre-catalysts bearing alternative ligands was tested to understand whether the poor reactivity of 2-chloropyridine could be overcome through selecting a different pre-catalyst. Unfortunately, [NiCl(*o*-tol)(PPh_3_)_2_], [NiCl(*o*-tol)(PCy_3_)_2_], [NiCl(*o*-tol)(dppe)], and [NiCl(*o*-tol)(XantPhos)] all produced only traces (<2% yield) of 2-phenylpyridine from attempted Suzuki–Miyaura cross-coupling reactions (Scheme 4(b)).

A series of stoichiometric reactions were also performed to assess the reactivity of **3-Cl** (Scheme 4(c)–(e)) This species does not react with excess phenylboronic acid and potassium phosphate, providing further support for our hypothesis that **3-Cl** is a stable off-cycle species. The stoichiometric reaction of **3-Cl** with PhSn(*n*-Bu)_3_ was conducted with the aim of establishing the viability of Stille cross-coupling reactions, but this simply returned complex **3-Cl**. However, the reaction of **3-Cl** with phenylmagnesium chloride led to an instantaneous colour change and the complete consumption of **3-Cl**.

**Scheme 5:**
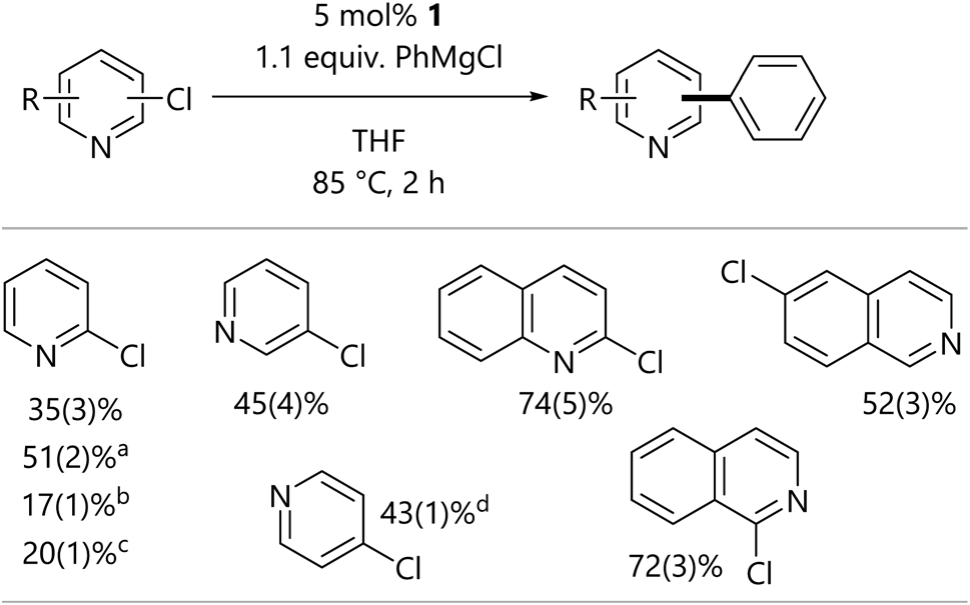
Kumada–Tamao–Corriu reactions of chloroheteroarenes. Results are quoted as conversion to product as determined by calibrated GC-FID analysis. ^a^Using 3 equiv. of PhMgCl for 4 h. ^b^Using 5 mol% **3-Cl** as a catalyst. ^c^Using 5 mol% **3-Cl** as a catalyst with 5 mol% of additional dppf. ^d^Used as the hydrochloride salt with 2.1 equiv. PhMgCl.

Consistent with the observations from stoichiometric experiments, the Kumada–Tamao–Corriu reactions of heteroaryl chlorides catalysed by **1** at 85 °C in THF led to successful catalytic turnover and the formation of 2-, 3-, and 4-phenylpyridine, 2- and 6-phenylquinoline, and 1-phenylisoquinoline (Scheme 5). These observations suggest that more reactive and more polar organometallic reagents are more capable of reacting with the dimeric butterfly complexes if and when they form during catalytic reactions. While this limits the substrate scope to substrates without protic or electrophilic functional groups, it still offers access to a range of compounds.

**Fig. 2:**
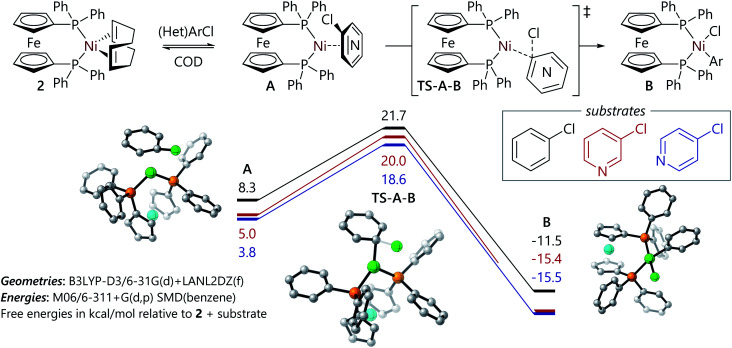
Free energy profiles for the reactions of chlorobenzene, 3-chloropyridine, and 4-chloropyridine with **2**. Images of **A**, **TS-A-B**, and **B** are provided for the representative example of the oxidative addition reaction of chlorobenzene.

**Fig. 3:**
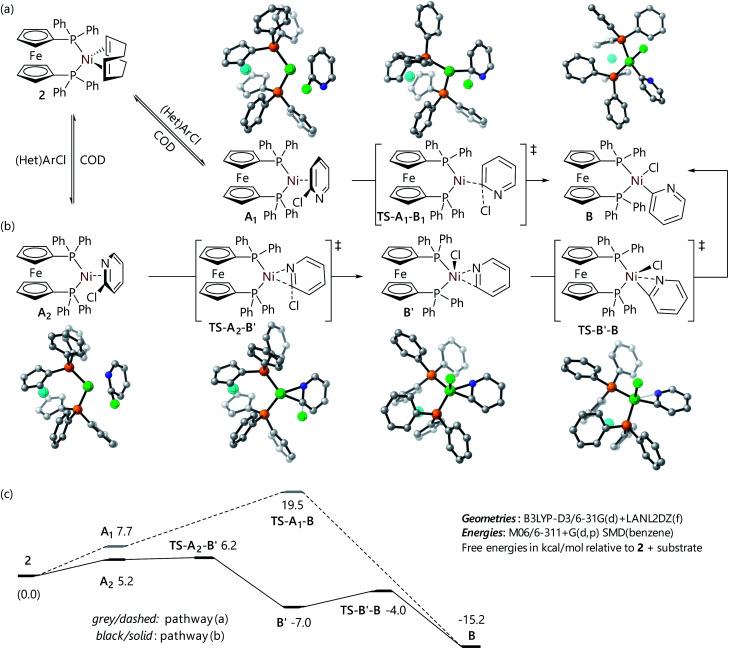
Structures for the reaction of 2-chloropyridine with **2***via* (a) an η^2^(C=C) complex and (b) an η^2^(C=N) complex, and (c) free energy profiles for these reactions.

The experimental work described above offers some insight into the poor reactivity of α-chloro-N-heterocycles in Suzuki–Miyaura reactions catalysed by dppf-nickel catalyst systems; DFT studies of this reaction mechanism were carried out to gain further insight into the reaction mechanism for the formation of the butterfly complexes, and to probe their electronic structure. Calculations were carried out with Gaussian 16 (ref. 31) using M06/6-311+G(d,p)/SMD (benzene)//B3LYP-D3/6-31G(d)+LANL2TZ(f) as described previously for similar systems;^32^ full details can be found in the ESI.† All energies are quoted as free energies in benzene solution relative to [Ni(dppf)(COD)] (**2**). Free energy profiles and selected structures are displayed in Fig. 2 and 3. Coordinates and energies for these structures can be obtained from the ioChem-BD data repository at http://dx.doi.org/10.19061/iochem-bd-6-112.

The stability of the ‘butterfly’ complex was assessed *versus* the corresponding [Ni(2-py)Cl(L)_*n*_] complex (*i.e.* the putative oxidative addition complex) for a small set of ligands: dppf, PPh_3_, PCy_3_, dppe, and Xantphos. These have a range of steric and electronic properties. Table 2 records the free energy of the oxidative addition products and the butterfly complexes for each ligand system *versus* [Ni(COD)_2_]. The oxidative addition complexes typically have G_rel_ of *ca.* −19 to −25 kcal mol^−1^ (except for the dppe system), while the butterfly complexes have G_rel_ of *ca.* −46 to −62 kcal mol^−1^. These data do not, of course, provide any information about how fast the latter complexes might form, but demonstrates that they are potentially thermodynamically stable off-cycle species for catalyst systems that have ligands other than dppf.

**Table 2:** Free energies of the oxidative addition complexes [Ni(2-py)Cl(L)_*n*_] and butterfly complexes (kcal mol^−1^ in benzene solution) *versus* [Ni(COD)_2_] plus 2-chloropyridine and the corresponding ligand

	dppf	PPh_3_	PCy_3_	dppe	XantPhos
[NiCl(2-py)(L)_*n*_]	−24.8	−22.8	−18.9	−2.9	−21.2
Butterfly complex	−61.7	−56.5	−45.5	−55.0	−52.7

The free energy profiles for the reactions of chlorobenzene and 3- and 4-chloropyridine are relatively straightforward (Fig. 2). Similarly to the corresponding reaction of bromo-benzene with **2**,^32^ they proceed *via* ligand exchange to form intermediate [Ni(η^2^-ArCl)(dppf)] complexes (**A**); this is followed by an oxidative addition transition state (**A-B-TS**) that leads to a square planar [Ni(Ar)(Cl)(dppf)] complex (**B**). In most cases, this nickel(ii) complex then undergoes comproportionation to form a nickel(i) complex, as has been established previously.^14,20,33^ The reaction of 3-chloropyridine can proceed *via* two different isomers of **A**, differentiated by which two carbon atoms are coordinated to nickel, that present free energy profiles that are very similar in energy (within 0.6 kcal mol^−1^ for each structure); only the more favourable pathway is presented here. Overall, these data establish that the reactions of 3- and 4-chloropyridine with **2** present slightly lower barriers than the reaction of chlorobenzene with **2**; this is consistent with the higher rate observed experimentally for the oxidative addition reaction of 3-chloropyridine *versus* the corresponding reaction of chlorobenzene. Coordination of the substrate to nickel(0) *via* nitrogen or chlorine was less favourable than η^2^-coordination, with structures lying *ca.* 10 kcal mol^−1^ or 20 kcal mol^−1^ above **2** in free energy terms. We have previously shown that halide abstraction reactions between aryl halides and **2** are not competitive with the alternative oxidative addition pathway,^32^ and instead are likely to occur when a three-coordinate intermediate^15,34^ or a sterically-encumbered nickel(0) complex is the reactive species.^35^

The computational analysis of the corresponding reaction of 2-chloropyridine yielded two different pathways depending on the isomer of the η^2^-complex that is formed initially; this can either involve coordination to the carbon atoms at positions two and three, or coordination to the nitrogen atom and the carbon atom at position 2. The reaction of the η^2^(C^2^=C3) complex (**A1**) proceeds *via* an oxidative addition transition state of a similar structure to that depicted for chlorobenzene in Fig. 2 (**TS-A1-B**), albeit with a slightly lower barrier (Fig. 3(a)). However, the η^2^(C^2^=N) complex (**A2**) undergoes oxidative addition *via* a transition state in which there is a very short Ni⋯N distance (**TS-A2-B′**); this leads to a distorted square-based pyramidal nickel(ii) complex with an apical chloride ligand and a 2-pyridyl ligand coordinated through both the carbon and the nitrogen atoms (**B′**) (Fig. 3(b)). A further (low energy) transition state (**TS-B′-B**), in which the apical chloride moves into the P–Ni–P plane in tandem with an increase in the Ni–N distance, links **B′** to **B**. The lower barrier for 2-chloropyridine oxidative addition *versus* 3-chloropyridine oxidative addition is qualitatively consistent with the faster reaction of 2-chloropyridine that is observed experimentally (Fig. 3(c)).

**Fig. 4:**
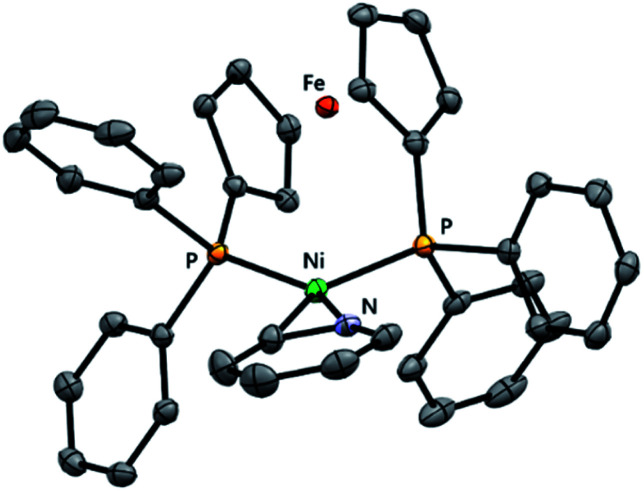
Molecular structure of the cation of **6** determined by single crystal X-ray diffraction analysis. Hydrogen atoms and the outer-sphere triflate counter ion are omitted for clarity.

A structure that is closely analogous to **B′** was obtained from the reaction of pyridin-2-yl triflate with **2**, as part of further (yet unsuccessful) attempts to overcome the apparent limitations of 2-chloropyridine in nickel-catalysed cross-coupling reactions (Fig. 4).^36^ [Ni(κ^2^-C,N-2-py)(dppf)][OTf] (**6**) features a geometry similar to that predicted from DFT calculations for **B′**, with a short Ni⋯N distance and a 2-pyridyl ligand in which the coordinated carbon and nitrogen atoms sit almost in the same plane as the two phosphorus atoms; the pyridine ring plane and the plane defined by P–Ni–P are 12.5° apart. The triflate counterion adopts a position away from the inner sphere, rendering this an ion pair and the nickel centre planar.

Further analysis was carried out to probe the electronic structure of the oxidative addition transition states. This analysis did not reveal any bond critical points between nickel and chlorine in the transition states, and therefore these reactions proceed *via* a concerted S_N_Ar-type oxidative addition rather than a three-centred oxidative addition. For the reactions of chlorobenzene and 2-chloropyridine with **2** the QTAIM analysis was carried out with the specific aim of establishing whether there is a significant N–Ni interaction during the oxidative addition reaction. QTAIM analysis was carried out using the AIMAll software program using extended wavefunction files obtained from single-point calculations on the optimised geometries.^37^

**Fig. 5:**
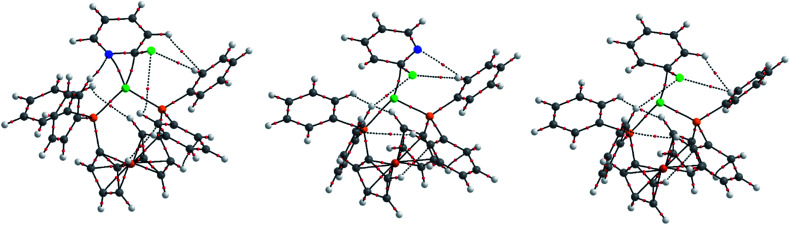
QTAIM analyses for oxidative addition transition states **TS-A2-B′** for 2-chloropyridine (left), **TS-A1-B** for 2-chloropyridine (middle), and **TS-A-B** for chlorobenzene (right). Red points represent bond critical points, while coloured spheres represent atomic positions (white = H; grey = C; light orange = P; light green = Cl; dark green = Ni; dark orange = Fe).

As expected, this analysis showed a bond critical point (BCP) between the nitrogen atom and the nickel centre in **TS-A2-B** (Fig. 5(a)), indicative of an interaction between the nitrogen atom and the nickel centre. In contrast, the alternative oxidative addition transition state for 2-chloropyridine (**TS-A1-B**) (Fig. 5(b)) and the oxidative addition transition state for chlorobenzene (**TS-A-B**) (Fig. 5(c)) do not show this interaction; instead, these show an interaction between the nickel centre and the *ipso* carbon atom only.

**Fig. 6:**
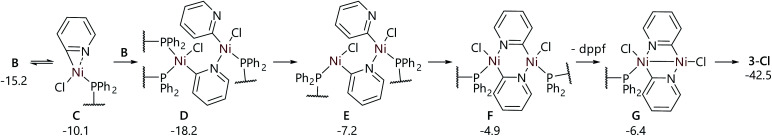
Proposed reaction mechanism for the formation of **3-Cl** from **B**. Energies are free energies in kcal mol^−1^ with respect to **2**, and are obtained from M06/6-311+G(d,p) single point calculations in benzene solvent (SMD) using B3LYP-D3/6-31G(d)+LANL2TZ(f) geometries.

A series of calculations was carried out to assess a plausible mechanism for the formation of **3-Cl** from **2**. This essentially involves a series of ligand exchanges and so no transition states were located, but a series of plausible intermediates were obtained (Fig. 6). This pathway proceeds *via* the de-coordination of one phosphorus atom from complex **B** derived from **2** and 2-chloropyridine; the nitrogen atom of the 2-pyridyl ligand occupies the vacant coordination site that is then generated, forming complex **C**. This can then dimerise with a second molecule of **B** to form **D**, in which the nickel centres are linked by a single 2-pyridyl unit, and then, *via***E**, complex **F** in which the centres are bridged by both pyridyl units. Dissociation of a dppf ligand yields **G**, which can then form **3-Cl**; this complex, plus one equivalent of dppf and two equivalents of COD, is 42.5 kcal mol^−1^ lower in free energy than two equivalents of **2** plus two equivalents of 2-chloropyridine. These data are consistent with the apparent role of **3-Cl** as a low energy off-cycle species that can only be engaged in a catalytic cycle if it undergoes transmetalation with a sufficiently nucleophilic organometal reagent. QTAIM analysis of **3-Cl** indicated only four BCPs involving nickel: these represent bonds to the phosphorus, chlorine, carbon, and nitrogen atoms only (see the ESI†). This strongly suggests that there is no direct bonding between the two nickel centres in these butterfly complexes, and that these are held in close proximity only by the bridging pyridine and dppf ligands and not by nickel–nickel interactions.

## Conclusions

We have identified and characterised a series of dinickel(ii) complexes that are formed from the reaction of α-halo-N-heteroarenes with [Ni(COD)(dppf)] (**2**). The same complexes arise during catalytic reactions where the related nickel(ii) pre-catalyst, [NiCl(*o*-tol)(dppf)], is used. These form rapidly; the oxidative addition of chloroheteroarenes to dppf-nickel(0) is found to be significantly faster than the corresponding reaction of aryl halides.

The dinickel(ii) complexes are not competent (pre)-catalysts for Suzuki–Miyaura reactions due to their poor reactivity with boronic acids and base. More polar organometallic species, such as Grignard reagents, will undergo reaction with the butterfly complexes and therefore Kumada–Tamao–Corriu reactions with this class of substrates are competent. It is anticipated that optimisation of the reaction conditions can further increase the yields of these reactions.

It must be noted that other (pre-)catalyst systems, reported by others working in the field of nickel catalysis, have been used to effect the Suzuki–Miyaura cross-coupling reactions of 2-chloropyridine and related compounds. These include studies that report single examples prepared using [NiCl_2_(dppp)] or [NiCl_2_(PCy_3_)_2_].^10,11^ However, Ge and Hartwig have used [NiCl(η^3^-cinnamyl)(dppf)] to deliver a range of compounds in high yields;^12^ this complex uses the same ancillary ligand (dppf) as was deployed in this study, but we note that all of the examples in this previous study deploy 2-(benzo)furanyl and 2-(beno)thiophenylboronic acids in a more coordinating solvent (acetonitrile). It may be the case that these are more nucleophilic, or that the coordination of the heteroatom or heterocycle enables these cross-coupling reactions to proceed. It has recently been shown by He *et al.* that nickel will coordinate to thiophenes in an η^2^ manner, and will even undergo reversible insertion into the C–S bond.^38^

Further work is underway in our laboratories to more fully understand reaction mechanisms and structure/reactivity relationships in the cross-coupling reactions of haloheteroarenes catalysed by nickel, with the aim of enabling the robust and reliable cross-coupling reactions of a wide range of substrates.

## Data availability

Data underpinning the experimental component of this publication are openly available from the University of Strathclyde Knowledgebase at https://doi.org/10.15129/a7c7ed8e-bb39-4632-8716-39d43c872264. Crystal structure data can be obtained from the CCDC at https://www.ccdc.cam.ac.uk/structures/ using deposition numbers 2103814 (**3-Cl**), 2103815 (**3-Br**), 2103816 (**5**), and 2103817 (**6**). Data underpinning computational work can be obtained from the ioChem-BD^39^ database hosted by the Barcelona Supercomputing Institute at .

## Author contributions

AKC: data curation, formal analysis, investigation, methodology; MEG: data curation, formal analysis, investigation, methodology, writing – review and editing; WD: formal analysis, investigation, methodology; PMB: project administration, supervision, writing – review and editing; TOR: project administration, supervision; ARK: data curation, formal analysis, investigation, methodology, validation, writing – review and editing; DJN: conceptualisation, funding acquisition, methodology, project administration, supervision, writing – original draft, writing – review and editing, formal analysis, investigation.

## Conflicts of interest

There are no conflicts to declare.

## Supplementary Material

SC-012-D1SC04582B-s001

SC-012-D1SC04582B-s002

SC-012-D1SC04582B-s003
