# Supplementary material for: Inhibition of (dppf)nickel-catalysed Suzuki–Miyaura cross-coupling reactions by α-halo-N-heterocycles
Source: Chem Sci. 2021 Oct 11;12(42):14074–82. doi: 10.1039/d1sc04582b (PMC8565371; doi:10.1039/d1sc04582b)
Supplement: SC-012-D1SC04582B-s001 [file SC-012-D1SC04582B-s001.pdf]

Electronic Supplementary Material (ESI) for Chemical Science.
This journal is © The Royal Society of Chemistry 2021

SUPPORTING INFORMATION FOR

Inhibition of (dppf)nickel-catalysed Suzuki-Miyaura cross-coupling reactions by α-halo-*N*-heterocycles

Alasdair K. Cooper,[a] Megan E. Greaves,[a,b] William Donohoe,[a] Paul M. Burton,[c] Thomas O. Ronson,[b] Alan R. Kennedy,[a] and David J. Nelson[a]

a) WestCHEM Department of Pure & Applied Chemistry, University of Strathclyde, 295 Cathedral Street, Glasgow, G1 1XL, Scotland
b) Chemical Development, Pharmaceutical Technology and Development, Operations, AstraZeneca, Macclesfield, SK10 2NA, UK
c) Syngenta, Jealott's Hill International Research Centre, Bracknell, Berkshire, RG42 6EY, UK

CONTENTS

Experimental Information ... 2
Synthesis ... 2
Stoichiometric Reactions of 3-Cl ... 9
  Procedure ... 9
  Outcomes ... 9
X-Ray Crystallography ... 11
GC-FID Calibration ... 13
Cross-Coupling Reactions ... 13
  Suzuki-Miyaura Cross-Coupling Procedure ... 13
  Kumada-Tamao-Corriu Cross-Coupling Procedure ... 13
  Reaction Outcomes ... 14
Kinetic Studies ... 18
  General Procedure ... 18
  Pseudo-first Order Rate Constants ... 18
  Kinetic Data Plots ... 19
  Spectra from Reactions of 2-chloropyridine ... 22
Computational Studies ... 23
  General ... 23
  Energies ... 23
  QTAIM Analysis ... 26
References ... 28

Experimental Information

General.

All reactions were carried out under an argon or nitrogen atmosphere using glovebox or Schlenk techniques in dry, degassed solvent unless otherwise stated.

Materials.

Anhydrous THF and toluene (<10 ppm water) were obtained from an Innovative Technologies PureSolv apparatus. [NiCl(*o*-tol)(dppf)][1] and [Ni(COD)(dppf)][2] were prepared according to literature methods. All other compounds, with the exception of those noted below, were obtained from commercial sources and used as supplied. [Ni(COD)$_2$] was stored in a glovebox freezer at -35 °C. Deuterated solvents for NMR kinetics were obtained from commercial suppliers and dried on 4 Å molecular sieves before use.

Analysis.

NMR spectroscopy was performed using either: a Bruker AV3-400 with liquid nitrogen cryoprobe, a Bruker AV3-500HD with BBFO SmartProbe, or a Bruker AVII-600 with BBO-*z*-ATMA probe. All kinetic studies were performed using the latter two instruments. ^{1}H NMR spectra were referenced to residual solvent peaks, ^{13}C{^{1}H} NMR spectra were referenced to solvent peaks, and ^{31}P{^{1}H} NMR spectra were externally referenced. Coupling constants are reported in Hertz.

GC-FID analysis was performed using an Agilent 7890A gas chromatograph fitted with an Agilent HP5 column (30 m x 0.25 mm I. D. x 0.25 µm). GC-MS analysis was performed using an Agilent 7890A gas chromatograph fitted with a RESTEK Rxi-5Sil column (30 m x 0.32 mm I. D. x 0.25 µm) and interfaced to an Agilent 5975 MSD running in EI mode.

Synthesis

General Procedure for the Synthesis of Butterfly Complexes. A Schlenk flask equipped with a stir bar was charged with [Ni(COD)$_2$] (1 equiv.) and dppf (1 equiv.) in an argon-filled glovebox. Anhydrous toluene was added and the reaction was stirred at room temperature for 2 h, during which time a [Ni(COD)(dppf)] precipitated. The corresponding heteroaryl halide (5 equiv.) was added and the mixture was stirred at room temperature overnight. The work-up was conducted under air; the solid product was filtered on a sintered glass frit (under air), washed with diethyl ether, and dried.

[{NiCl(μ-2-py)}$_2$(dppf)] (3-Cl). Synthesised according to the general procedure using [Ni(COD)$_2$] (100.1 mg, 0.363 mmol), dppf (201.7 mg, 0.363 mmol, 1 equiv.), and 2-chloropyridine (0.171 mL, 1.815 mmol, 5 equiv.) in toluene (2 mL). Yield: 147 mg (90%) of a dark red powder.

^{1}H NMR (400 MHz, CDCl$_3$): δ_H 8.42 (s, 2H, Ar CH), 8.02 – 6.91 (m, 22H, Ar CH), 6.51 (s, 2H, Ar CH), 6.33 (s, 2H, Ar CH), 6.06 (s, 2H, ferrocene CH), 4.65 (s, 2H, ferrocene CH), 4.46 (s, 2H, ferrocene CH), 3.88 (s, 2H, ferrocene CH).

^{31}P{^{1}H} NMR (162 MHz, CDCl$_3$): δ_P 21.6 (s).

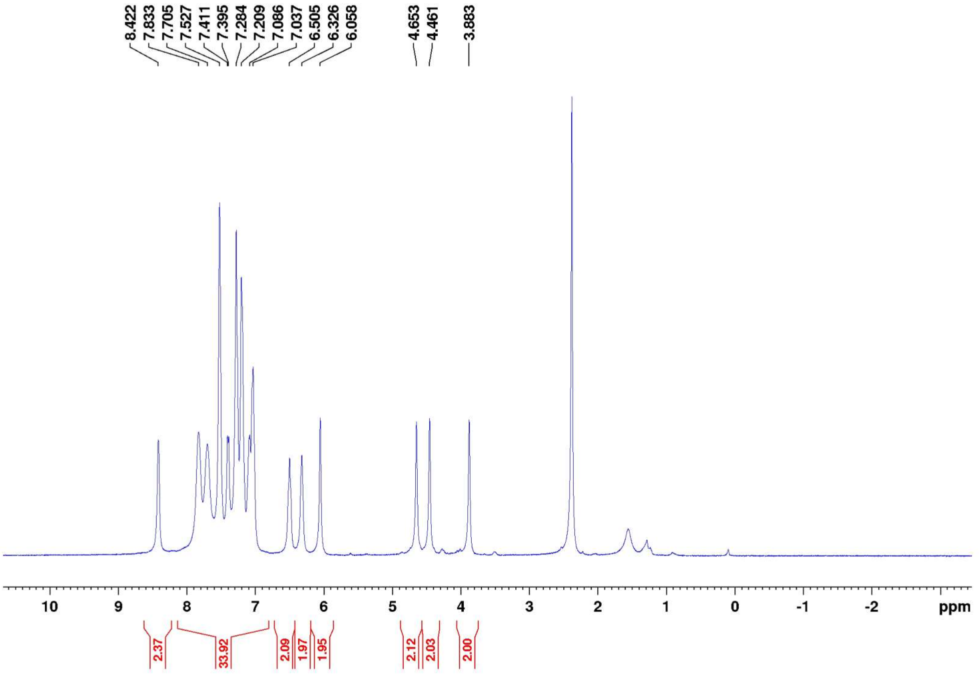

Figure S1. ^{1}H NMR spectrum of complex **3-Cl**.

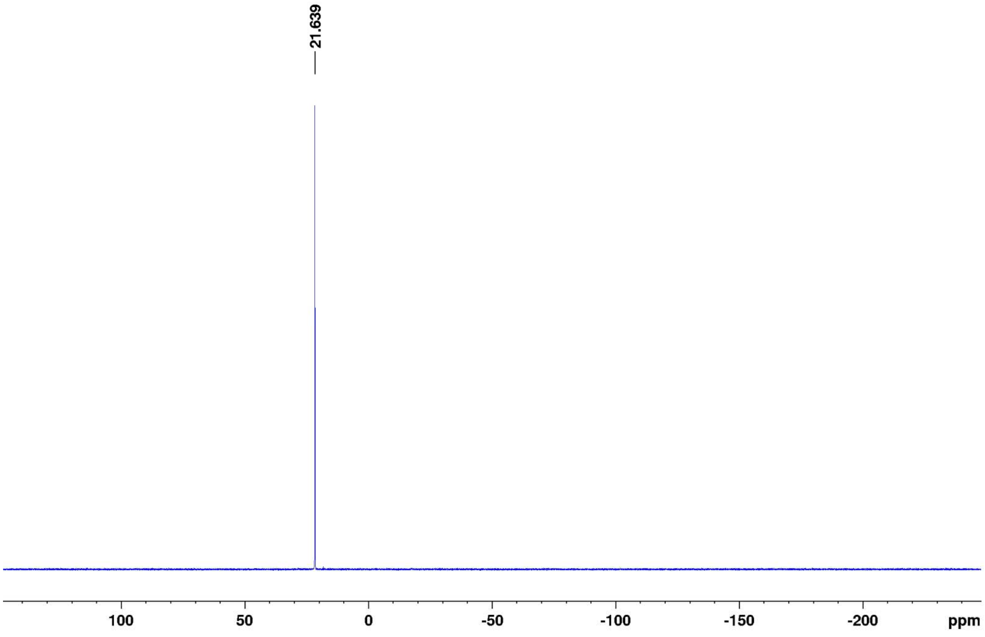

Figure S2. ^{31}P{^{1}H} NMR spectrum of complex **3-Cl**.

[{NiBr(μ-2-py)}$_2$(dppf)] (3-Br). Synthesised according to the general procedure using [Ni(COD)$_2$] (101.3 mg, 0.363 mmol), dppf (201.1 mg, 0.363 mmol, 1 equiv.), and 2-bromopyridine (0.174 mL, 1.815 mmol, 5 equiv.) in toluene (2 mL). Yield: 98.1 mg (54%) of a brown-red powder.

^{1}H NMR (400 MHz, CDCl$_3$): δ_H 8.62 (s, 2H, Ar CH), 7.74 (s, 8H, Ar CH), 7.52 (s, 6H, Ar CH), 7.44 (d, 2H, Ar CH, *J* = 8.0), 7.08 (s, 6H, Ar CH), 6.52 (s, 2H, Ar CH), 6.30 (2H, Ar CH), 6.14 (s, 2H, Fc CH), 4.69 (s, 2H, Fc CH), 4.49 (s, 2H, Fc CH), 3.90 (s, 2H, Fc CH).

^{31}P{^{1}H} NMR (162 MHz, CDCl$_3$): δ_P 22.5 (s).

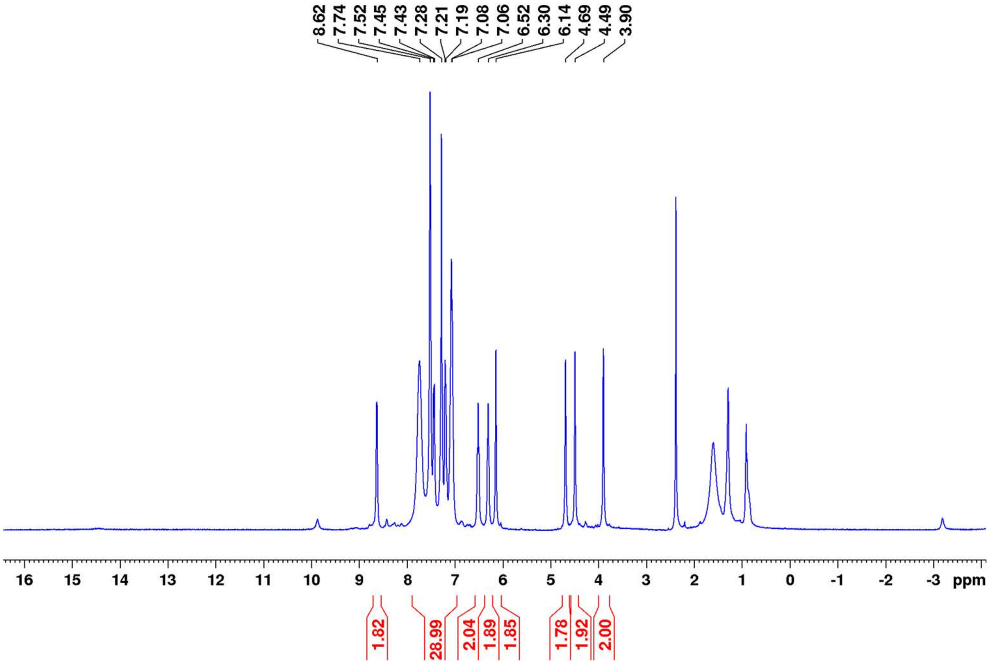

Figure S3. ^{1}H NMR spectrum of complex **3-Br**.

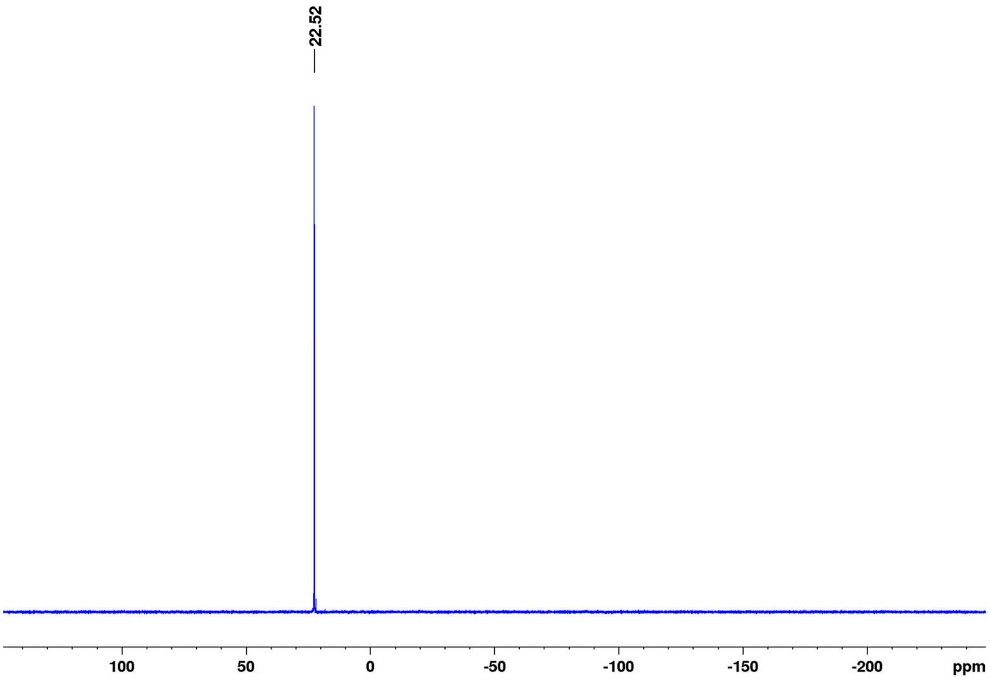

Figure S4. ^{31}P{^{1}H} NMR spectrum of complex **3-Br**.

[{NiCl(μ-2-quin)}$_2$(dppf)] (4). Synthesised according to the general procedure using [Ni(COD)$_2$] (102.3 mg), dppf (202.4 mg, 0.363 mmol, 1 equiv.), and 2-chloroquinoline (297.1 mg, 1.815 mmol, 5 equiv.) in toluene (2 mL). Yield: 75.1 mg (42%) of a dark red powder.

^{1}H NMR (400 MHz, $CDCl_3$): δ_H 9.12 (d, 2H, Ar CH, J = 8.5), 8.13 (app. br. s, 4H, Ar CH), 7.84 (app. br. s, 4H, Ar CH), 7.70 – 7.50 (m, 10H, Ar CH), 7.20 – 7.18 (m, 4H, Ar CH), 7.01 – 6.81 (m, 8H, Ar CH), 6.19 (s, 2H, Fc CH), 4.63 (s, 2H, Fc CH), 4.42 (s, 2H, Fc CH), 3.93 (s, 2H, Fc CH).

^{31}P{^{1}H} NMR (162 MHz, $CDCl_3$): δ_P 22.6 (s).

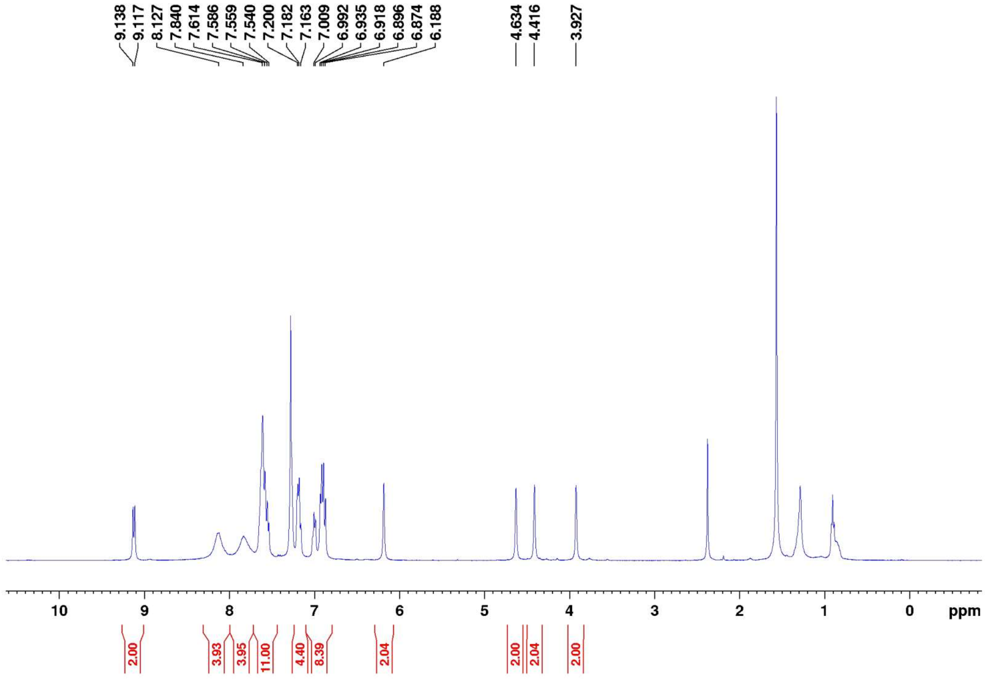

Figure S5. ^{1}H NMR spectrum of complex **4**.

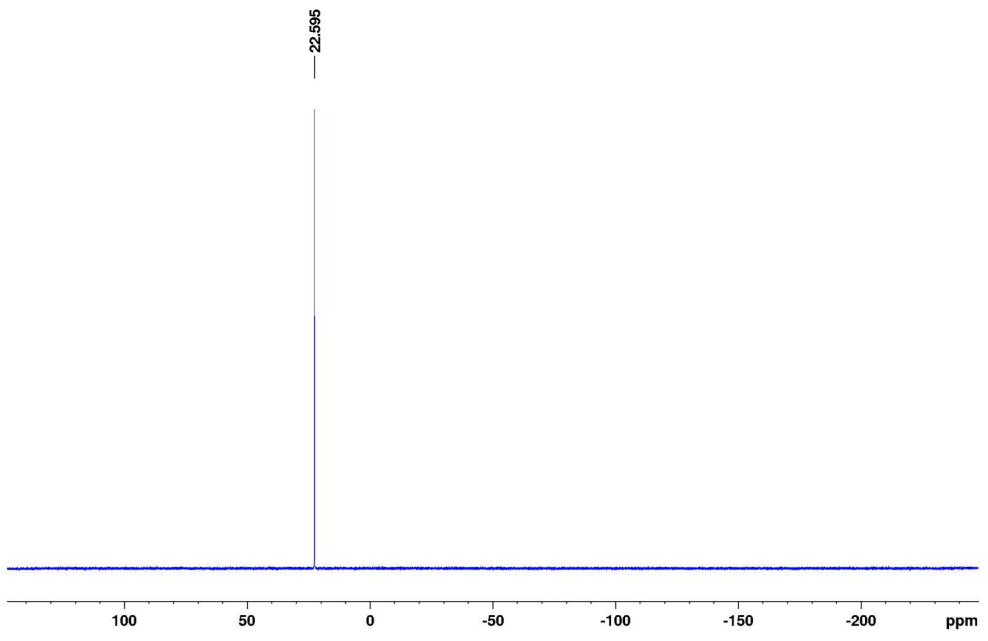

Figure S6. ^{31}P{^{1}H} NMR spectrum of complex **4**.

[{NiCl(μ-2-isoquin)}$_2$(dppf)] (5). Synthesised according to the general procedure using [Ni(COD)$_2$] (100.6 mg, 0.363 mmol), dppf (201.2 mg, 0.363 mmol, 1 equiv.), and 1-chloroisoquinoline (297.4 mg, 1.815 mmol, 5 equiv.) in toluene (2 mL). Yield: 21 mg (12%) of a dark red powder.

^{1}H NMR (400 MHz, CDCl$_3$): δ_H 10.20 (d, 2H, Ar CH, *J* = 8.2), 8.36 (dd, 2H, Ar CH, *J* = 6.6, 1.8), 8.15 – 8.04 (m, 4H, Ar CH), 7.52 – 7.41 (m, 10H, Ar CH), 7.36 (t, 2H, Ar CH, *J* = 7.4), 7.23 – 7.17 (m, 2H, Ar CH), 7.15 (m, 2H, Ar CH, *J* = 7.9), 6.84 (t, 2H, Ar CH, *J* = 7.4), 6.72 (td, 4H, Ar CH, *J* = 7.8, 2.2), 6.57 (d, 2H, Ar CH, *J* = 6.5), 6.10 (d, 2H, Fc CH, *J* = 1.1), 4.58 (s, 2H, Fc CH), 4.42 (d, 2H, Fc CH, *J* = 1.0), 4.24 (s, 2H, Fc CH).

^{31}P{^{1}H} NMR (162 MHz, CDCl$_3$): δ_P 18.2 (s).

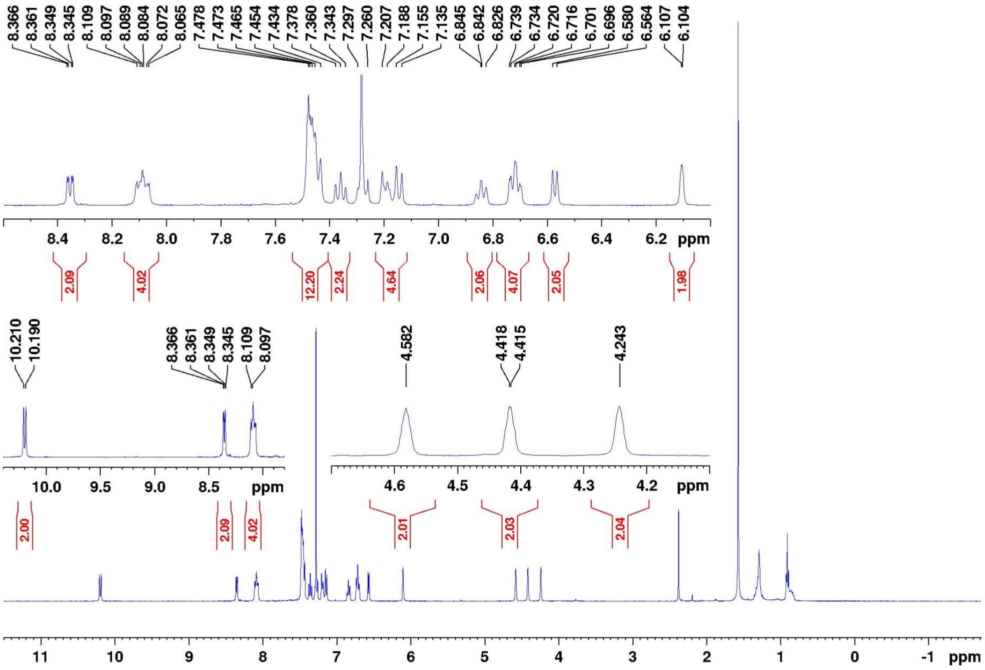

Figure S7. ^{1}H NMR spectrum of complex **5**.

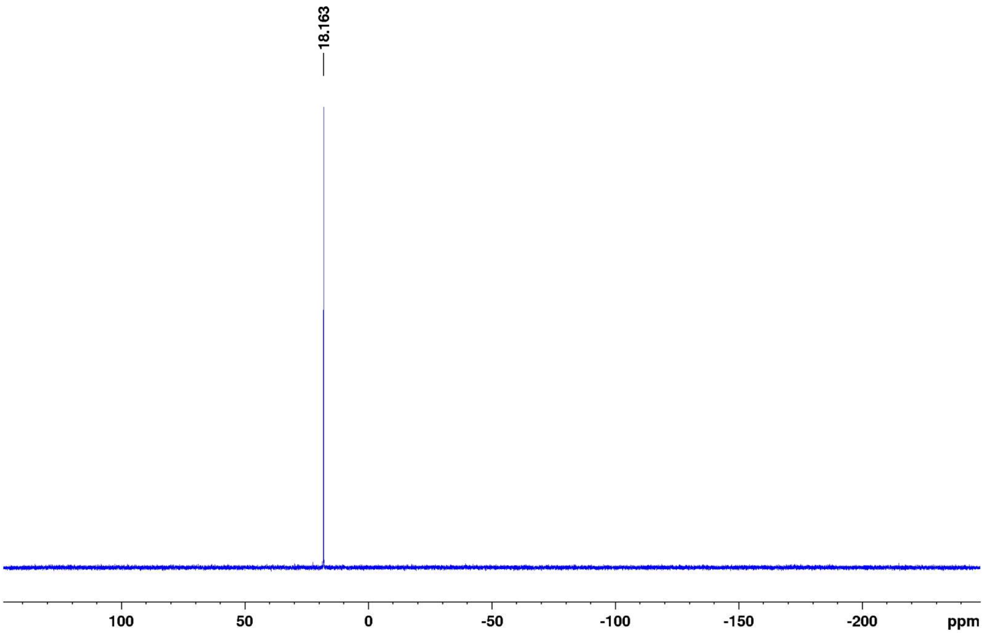

Figure S8. ^{31}P{^{1}H} NMR spectrum of complex **6**.

6-Phenylquinoline. Prepared according to the literature procedure.[3] In the glovebox, palladium(II) acetate (14 mg, 0.062 mmol), XPhos (35 mg, 0.074 mmol), 6-chloroquinoline (0.5 g, 3.1 mmol), and phenylboronic acid (0.44 g, 3.7 mmol) were added to a microwave vial equipped with a large stir bar. Outside the glovebox, degassed *n*-butanol (17 mL) and a solution of caesium hydroxide hydrate (0.46 g, 5.3 mmol) in degassed water (0.4 mL) were added. The reaction was stirred vigorously at 30 °C overnight. The phases were then separated and the aqueous phase was extracted with ethyl acetate (3 x 20 mL). The organic extracts were combined, dried over magnesium sulfate, filtered, and concentrated under reduced pressure. The crude product was purified *via* column chromatography on silica gel. Yield: 0.3 g (47%) of a pale-yellow solid.

^{1}H NMR (400 MHz, $CDCl_3$): δ_H 9.00 – 8.99 (m, 1H), 8.01 – 7.94 (m, 2H), 7.76 – 7.74 (m, 2H), 7.61 – 7.42 (m, 6H).

^{13}C{^{1}H} NMR (101 MHz, $CDCl_3$): δ_C 149.7, 146.9, 139.8, 138.9, 136.1, 134.8, 131.6, 129.3, 128.9, 128.5, 127.4, 127.3, 127.0, 125.0, 121.0.

m/z (GC-MS, EI): 205.2.

NMR data are consistent with the literature.[3]

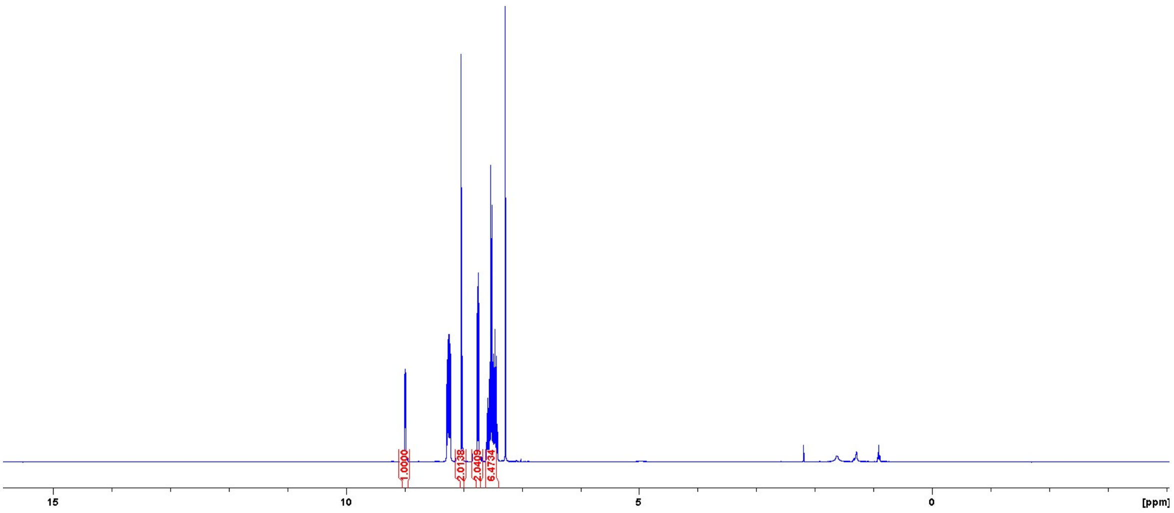

Figure S9. ^{1}H NMR spectrum of 6-phenylquinoline.

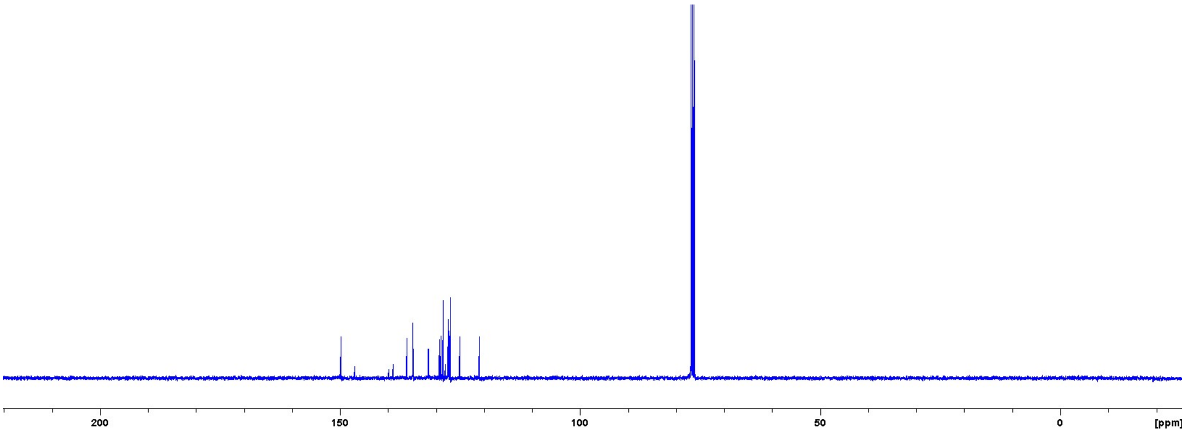

Figure S10. ^{13}C{^{1}H} NMR spectrum of 6-phenylquinoline.

Pyridin-2-yl triflate. Prepared according to a literature procedure.[4] 2-Hydroxypyridine (1 g, 10.5 mmol) was dissolved in toluene (40 mL). A solution of potassium phosphate (30% *w/w* in water, 40 mL) was added and the mixture was cooled in an ice bath. Trifluoromethanesulfonic anhydride (2.1 mL, 12.6 mmol) was added dropwise and the reaction was stirred at room temperature for 30 min. The layers were separated and the organic layer was washed with water. The solvent was removed and the crude product was purified by column chromatography on silica gel. Yield: 1.2 g (50%) of a colourless oil.
^{1}H NMR (400 MHz, $CDCl_3$): δ_H 8.43 (dd, *J* = 4.7, 2.1, 1H), 7.93 (td, *J* = 7.8, 2.1, 1H), 7.41 (dd, *J* = 7.4, 4.8, 1H), 7.21 (d, *J* = 8.2, 1H).
^{13}C{^{1}H} NMR (101 MHz, $CDCl_3$): δ_C 155.5, 148.3, 140.5, 123.7, 118.1 (q, $J_{C\text{-}F}$ = 321.3), 114.8.
^{19}F{^{1}H} NMR (376 MHz, $CDCl_3$): δ_F -73.2.
m/z (GC-MS, EI): 227.0.
NMR data are consistent with the literature.[5]

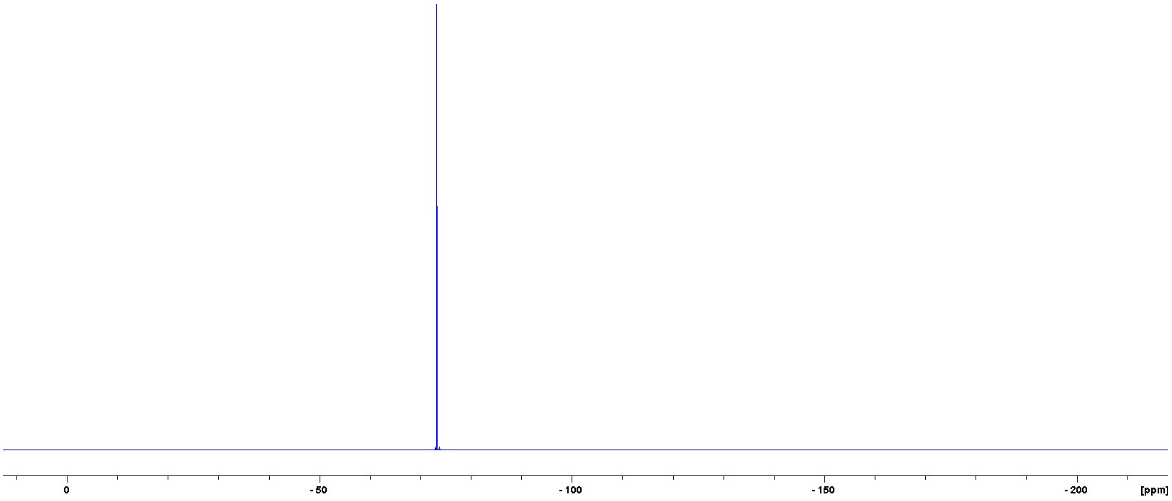

Figure S11. ^{19}F{^{1}H} NMR spectrum of pyridin-2-yl triflate.

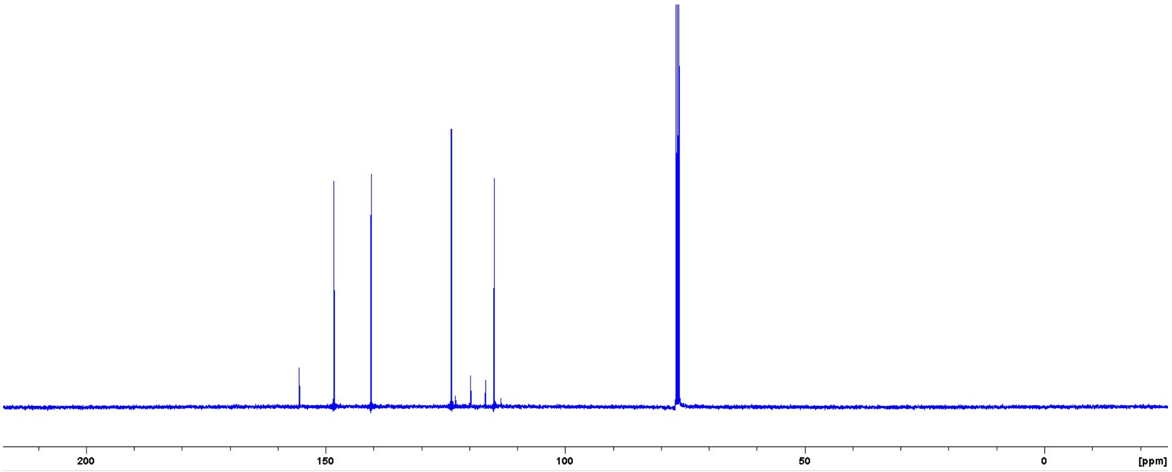

Figure S12. ^{13}C{^{1}H} NMR spectrum of pyridin-2-yl triflate.

Stoichiometric Reactions of 3-Cl

Procedure

A sample of **3-Cl** was dissolved in anhydrous THF and an excess of an organometallic reagent was added: 5 equiv. PhMgCl, PhSn(*n*-Bu)$_3$, or PhB(OH)$_2$/K_3PO_4; in the latter case, the solvent mixture was 4:1 *v*/*v* THF/water. The reactions were stirred at room temperature for 24 h. An aliquot was diluted in chloroform and analysed by GC-FID to check for the presence of 2-phenylpyridine by comparison with the retention time of an authentic sample. The reactions with PhMgCl and PhSn(*n*-Bu)$_3$ were also analysed by ^{31}P{^{1}H} NMR spectroscopy by taking a sample and diluting in chloroform-*d*.

Outcomes

(i) *PhMgCl*: 2-phenylpyridine was detected by GC-FID. The ^{31}P{^{1}H} NMR spectrum indicated the presence of an as-yet unidentified new complex (δ_P = 18.1 ppm) (Figure S13).

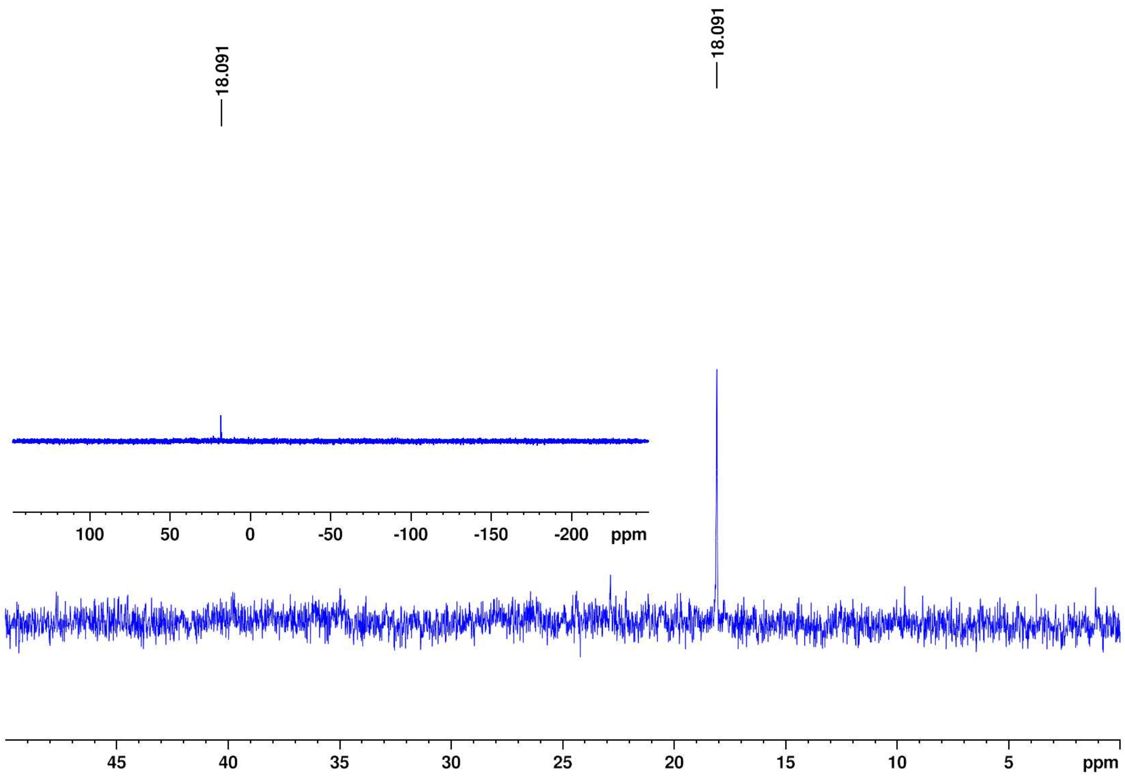

Figure S13. ^{31}P{^{1}H} NMR spectrum from the reaction of **3-Cl** with phenylmagnesium chloride.

(ii) *PhSn(n-Bu)$_3$:* No 2-phenylpyridine was detected by GC-FID. The $^{31}P\{^1H\}$ NMR spectrum indicated that only **3-Cl** was present (Figure S14).

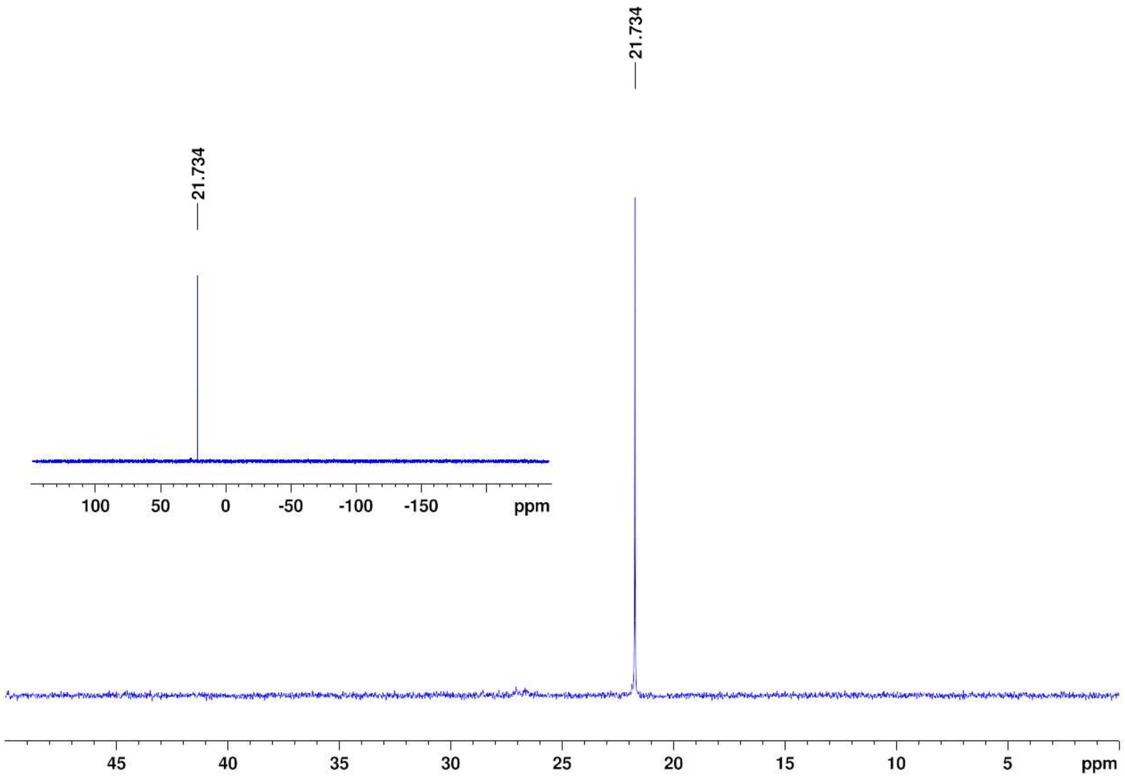

Figure S14. $^{31}P\{^1H\}$ NMR spectrum from the attempted reaction of **3-Cl** with pheny(tributyl)tin.

(iii) *PhB(OH)$_2$/K$_3$PO$_4$:* No 2-phenylpyridine was detected by GC-FID.

X-Ray Crystallography

Single crystal x-ray diffraction data for **3-Cl** and **3-Br** were measured with an Oxford Diffraction Gemini S instrument while data for **5** and **10** were measured with a Rigaku Synergy-i instrument. All used Cu Kα(λ = 1.54184 Å) radiation. Data collection and processing used CrysalisPro software.[6]

The structures were refined to convergence on F^2 using all independent reflections and the program SHELXL-2018 as implemented within WinGX.[7, 8] The non-hydrogen atoms were refined anisotropically and hydrogen atoms were placed in idealised positions and refined in riding modes.

For **3-Br** one dichloromethane solvate molecule was modelled as disordered over two sites.

For **6**, the deprotonated pyridine ligand was modelled over two orientations – effectively swapping the N and C binding sites of Ni. The two orientations of the ligand were found to have essentially equal site occupancies.

Selected crystallographic data and refinement parameters are presented below. CCDC deposition numbers CCDC 2103814 to 2103817 contain the full supplementary crystallographic data for this paper in cif format. These data are provided free of charge by the joint Cambridge Crystallographic Data Centre and Fachinformationszentrum Karlsruhe Access Structures service www.ccdc.cam.ac.uk/structures.

Table S1. Selected crystallographic data and refinement parameters.

**Compound**	**3-Cl**	**3-Br**	**5**	**6**
**CCDC**	2103814	2103815	2103816	2103817
**Formula**	$C_{56}H_{48}Cl_2$ $FeN_2Ni_2P_2$	$C_{46}H_{40}Br_2$ $Cl_4FeN_2Ni_2P_2$	$C_{52}H_{40}Cl_2$ $FeN_2Ni_2P_2$	$C_{40}H_{32}F_3$ $FeNNiO_3P_2S$
**Solvent**	2 x benzene	2 x CH_2Cl_2	none	none
**Form. Wt.**	1055.07	1157.63	998.97	840.22
**Space Group**	C2/c	$P2_1/n$	C2/c	P-1
**Crystal system**	Monoclinic	Monoclinic	Monoclinic	Triclinic
**Temp. (K)**	123(2)	123(2)	100(2)	100(2)
**a (Å)**	22.6705(3)	12.7154(1)	23.0991(3)	12.7001(2)
**b (Å)**	10.5061(2)	17.1989(2)	10.3327(1)	12.7903(2)
**c (Å)**	19.7987(3)	20.6947(2)	18.9251(3)	14.1264(2)
**α (°)**	90	90	90	93.421(1)
**β (°)**	90.4215(13)	95.5400(10)	106.3800(10)	114.030(1)
**γ (°)**	90	90	90	116.413(2)
**Volume (Å^3)**	4715.50(13)	4504.60(8)	4333.63(10)	1793.83(6)
**Z**	4	4	4	2
**Z'**	0.5	1	0.5	1
**Measured Reflections**	9541	34815	32177	31389
**Unique Reflections**	4646	8918	4296	6934
**2θmax (°)**	146.342	146.210	145.528	142.802
**R_{int}**	0.0285	0.0587	0.0626	0.0250
**Observed Reflections [I>2σI]**	4335	7957	4059	6767
**No. Parameters**	294	542	276	470
**S**	1.039	1.017	1.054	1.047
**R [on *F*, obs refls only]**	0.0343	0.0455	0.0408	0.0289
**ωR [on F^2, all data]**	0.0920	0.1255	0.1098	0.0744
**Largest diff. peak /hole (eÅ^{-3})**	0.428/-0.405	1.296/-0.926	0.835/-0.433	0.585/-0.476

GC-FID Calibration

The GC-FID apparatus was calibrated using authentic samples of each substrate and product by analysing a series of samples with known ratios of analyte to internal standard (*n*-dodecane). The response factors for each analyte are recorded in Table S2.

Table S2. Response factors for substrates and products used in this study.

**Analyte**	**Response Factor**	**Analyte**	**Response Factor**
Chlorobenzene	0.5342	4-Methyl-1,1-biphenyl	0.9947
2-Chloropyridine	0.3219	2-Phenylpyridine	0.7815
3-Chloropyridine	0.3328	3-Phenylpyridine	0.7110
4-Chloropyridine hydrochloride	0.3594	4-Phenylpyridine	0.6749
2-Chloroquinoline	0.6743	2-Phenylquinoline	0.9543
1-Chloroisoquinoline	0.6567	1-Phenylisoquinoline	0.9536
6-Chloroquinoline	0.6523	6-Phenylquinoline	0.7742

Cross-Coupling Reactions

Suzuki-Miyaura Cross-Coupling Procedure

A microwave vial equipped with a stirrer bar was charged with the aryl halide (if solid) (0.25 mmol), the catalyst (0.0125 mmol, 5 mol%), any additives, $PhB(OH)_2$ (0.28 mmol, 1.1 equiv.) and K_3PO_4 (0.75 mmol, 3 equiv.). The vial was capped and then evacuated and backfilled with nitrogen three times. A degassed mixture of THF/H_2O (4:1, 2 mL) was added, followed by the aryl halide (if liquid). The reaction was heated to 85 °C and stirred for 2 hours. After this time, the cap was pierced with a needle. Once cool, a known mass of dodecane was added, the reaction was filtered through celite and a sample was taken and diluted for GC-FID analysis.

Kumada-Tamao-Corriu Cross-Coupling Procedure

A microwave vial equipped with a stirrer bar was charged with the aryl halide (if solid) (0.25 mmol), the catalyst (0.0125 mmol, 5 mol%) and any additives. The vial was capped and then evacuated and backfilled with nitrogen three times. Anhydrous THF (1 mL), the aryl halide (if liquid) and PhMgCl (0.28 mmol, 1.1 equiv., as a solution in THF) were added. The reaction was heated to 85 °C and stirred for 2 hours. After this time, the cap was pierced with a needle. Once cool, a known mass of dodecane was added, the reaction was filtered through celite, and a sample was taken for GC-FID analysis.

Reaction Outcomes

Table S3. Duplicate results from Suzuki-Miyaura cross-couplings catalysed by [Ni(dppf)(o-tol)Cl]. Data for Scheme 1.

Entry	Substrate	Remaining Substrate (%)	Cross-coupled product (%)	Biphenyl (%)
1a	Chlorobenzene*	52	37	0
1b		51	38	0
2a	2-Chloropyridine	100	0	3
2b		100	0	3
3a	3-Chloropyridine	74	26	4
3b		74	26	4
4a	4-Chloropyridine hydrochloride	63	13	2
4b		65	13	3
5a	2-Chloroquinoline	79	0	2
5b		75	0	3
6a	1-Chloroisoquinoline	63	0	3
6b		78	0	2
7a	6-Chloroquinoline	46	29	4
7c		64	31	3

*Reaction carried out with *p*-tolylboronic acid

Table S4. Duplicate results from Suzuki-Miyaura cross-couplings reactions with 2-chloropyridine as the substrate catalysed by **3-Cl** with various additives. Data for Scheme 3.

**Entry**	**Additive (1.1 equiv.)**	**Remaining Substrate (%)**	**Cross-coupled product (%)**	**Biphenyl (%)**
1a	LiCl	100	0	0
1b		100	0	0
2a	$MgCl_2$	100	0	0
2b		98	0	0
3a	$ZnCl_2$	100	0	0
3b		100	0	0
4a	$AlCl_3$	100	0	0
4b		100	0	0

Table S5. Duplicate results from cross-coupling reactions with 2-chloropyridine as the substrate catalysed by **3-Cl** with and without added dppf.

**Entry**	**Cross-coupling Reaction**	**Additive (5 mol%)**	**Remaining Substrate (%)**	**Cross-coupled product (%)**	**Biphenyl (%)**
1a	Suzuki	None	98	2	0
1b			98	2	0
2a		dppf	97	3	0
2b			98	2	0
3a	Kumada	None	64	18	14
3b			67	16	13
4a		dppf	66	19	12
4b			65	21	17

Table S6. *Duplicate results from Kumada-Tamao-Corriu cross-coupling reactions catalysed by [Ni(dppf)(o-tol)Cl]. Data for Scheme 4.*

Entry	Substrate	Remaining Substrate (%)	Cross-coupled product (%)	Biphenyl (%)
1a	2-Chloropyridine	0	32	21
1b		18	37	33
2a	3-Chloropyridine	21	41	10
2b		24	49	11
3a	4-Chloropyridine hydrochloride	8	43	21
3b		2	42	29
4a	2-Chloroquinoline	6	79	9
4b		16	69	10
5a	1-Chloroisoquinoline	18	69	8
5b		9	74	8
6a	6-Chloroquinoline	13	54	19
6b		8	49	18
7a	2-Chloropyridine (with 1.5 equiv. PhMgCl)	0	41	*nd*
7b		0	41	*nd*
7c		0	42	*nd*
7d		0	40	*nd*
8a	2-Chloropyridine (with 3 equiv. PhMgCl, for 4 h)	0	49	*nd*
8b		0	51	*nd*
8c		0	52	*nd*
8d		0	51	*nd*

(*nd* = not determined)

***Table S7**. Results from the Suzuki-Miyaura cross-coupling reactions catalysed by alternative catalysts. All attempted reactions were between 2-chloropyridine and phenylboronic acid (with K_3PO_4) using the standard conditions noted above and in the manuscript.*

**Entry**	**Catalyst**	**Conversion to 2-phenylpyridine**
1	[NiCl(*o*-tol)(PPh_3)$_2$]	<1%
2	[NiCl(*o*-tol)(XantPhos)]	1%
3	[NiCl(*o*-tol)(PCy_3)$_2$]	<1%
4	[NiCl(*o*-tol)(dppe)]	<1%

Kinetic Studies

General Procedure

Kinetic data were obtained in the same manner as used previously, by following the decay of [Ni(COD)(dppf)] (**2**).[9-12] In an argon-filled glovebox, a septum-fitted NMR tube was charged with a solution of **2** (8 mg, 0.011 mmol) and dry benzene-d_6 or toluene-d_8 (0.5 mL). This sample was used to tune, match, lock, and shim the spectrometer, and to set the receiver gain. A spectrum was acquired to check the integrity of the sample. The aryl halide (20 equiv.) was added to start the reaction and $^{31}P\{^{1}H\}$ NMR spectra (16 scans per spectrum; 2 s between pulses) were acquired at intervals until >87% conversion (3$t_{1/2}$) was achieved. All experiments were performed in duplicate.

In each case, the absolute integral for **2** in each spectrum was normalised to that of the initial spectrum in order to obtain concentrations for those reactions. Rate constants were obtained by plotting the natural log of the concentration of **2** *versus* time.

Pseudo-first Order Rate Constants

Table S8. Rate constants obtained for substrates from plotting the natural log of the concentration of Ni^0 *versus* time. All reactions were carried out with [Ni(COD)(dppf)] (**2**) (22 mmol L^{-1}), 20 equivalents of substrate (0.44 mol L^{-1}) in 0.5 mL benzene-d_6 or toluene-d_8.

Entry	Substrate	Solvent	T (K)	k_{obs} (s^{-1})
1a	Chlorobenzene	Tol-d_8	343	4.01 x 10^{-3}
1b				3.34 x 10^{-3}
2a	Chlorobenzene	C_6D_6	323	2.50 x 10^{-4}
2b				2.68 x 10^{-4}
3a	2-Chloropyridine	C_6D_6	293	3.71 x 10^{-3}
3b				3.34 x 10^{-3}
4a	2-Chloropyridine	C_6D_6	283	1.17 x 10^{-3}
4b				1.08 x 10^{-3}
5a	3-Chloropyridine	C_6D_6	293	2.81 x 10^{-3}
5b				2.63 x 10^{-3}
6a	2-Chloroquinoline	C_6D_6	283	2.53 x 10^{-3}
6b				2.36 x 10^{-3}
7a	1-Chloroisoquinoline	C_6D_6	293	2.34 x 10^{-3}
7b				2.84 x 10^{-3}
8a	6-Chloroquinoline	C_6D_6	283	2.50 x 10^{-3}
8b				2.51 x 10^{-3}

Kinetic Data Plots

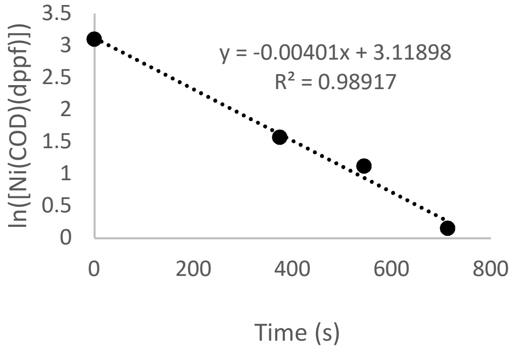

Figure S15. *Kinetic plot for Entry 1a, Table S6.*

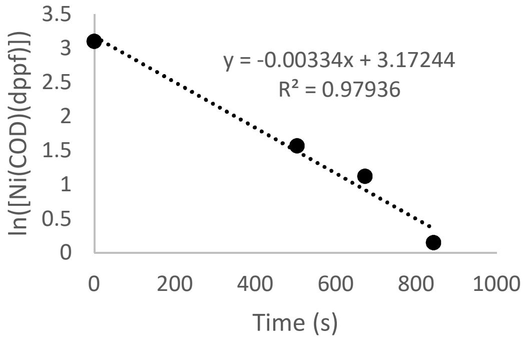

Figure S16. *Kinetic plot for Entry 1b, Table S6.*

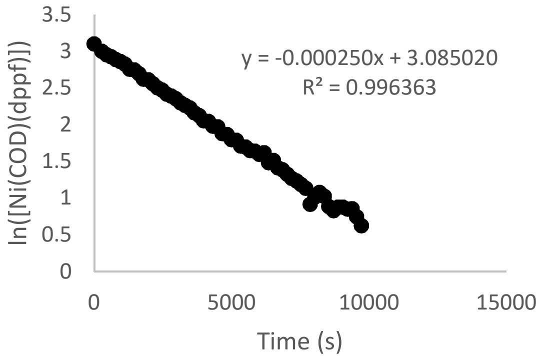

Figure S17. *Kinetic plot for Entry 2a, Table S6.*

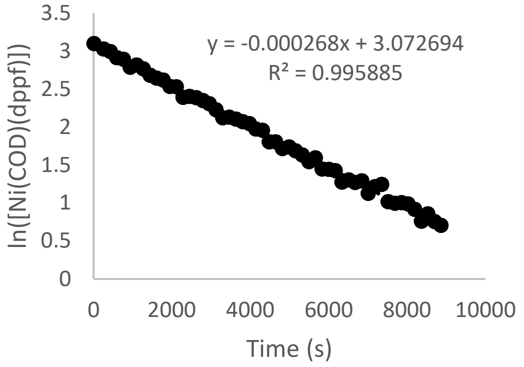

Figure S18. *Kinetic plot for Entry 2b, Table S6.*

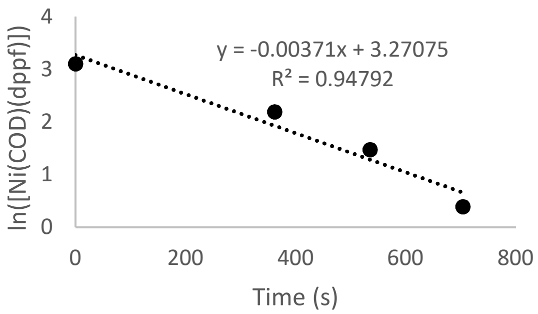

Figure S19. *Kinetic plot for Entry 3a, Table S6.*

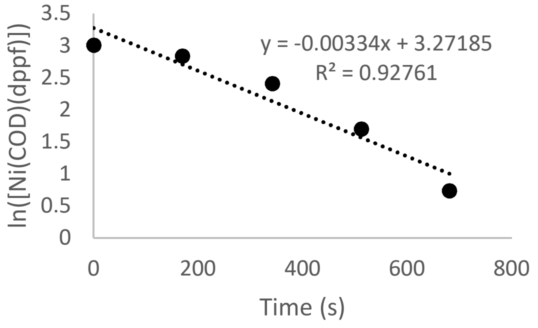

Figure S20. *Kinetic plot for Entry 3b, Table S6.*

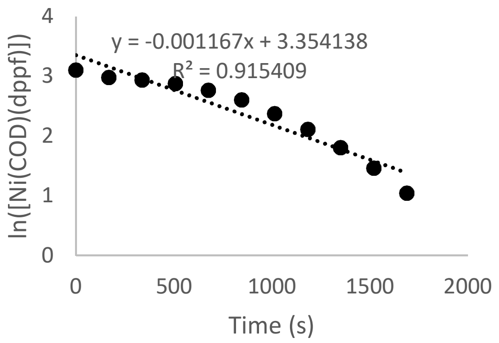

Figure S21. *Kinetic plot for Entry 4a, Table S6.*

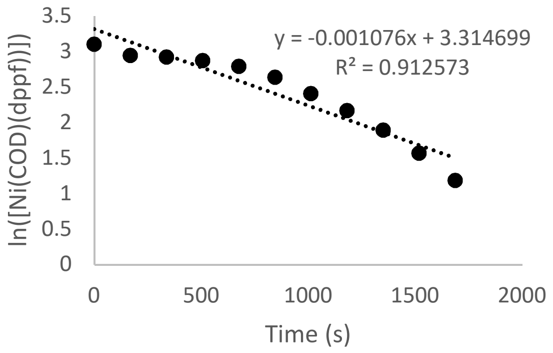

Figure S22. *Kinetic plot for Entry 4b, Table S6.*

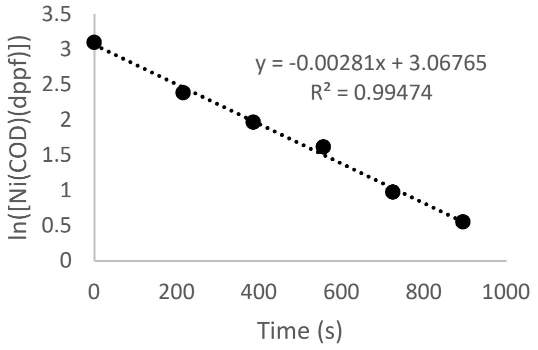

Figure S23. *Kinetic plot for Entry 5a, Table S6.*

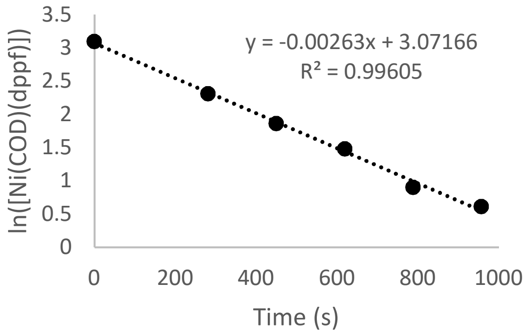

Figure S24. *Kinetic plot for Entry 5b, Table S6.*

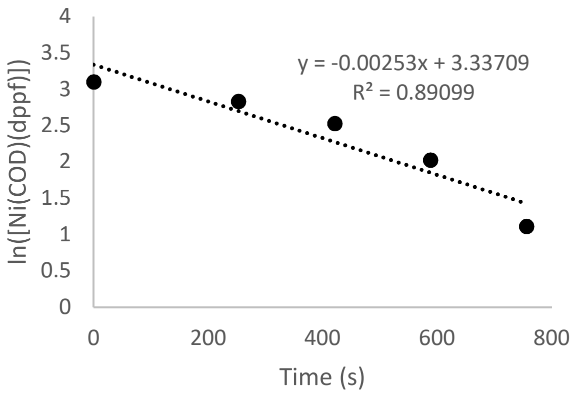

Figure S25. *Kinetic plot for Entry 6a, Table S6.*

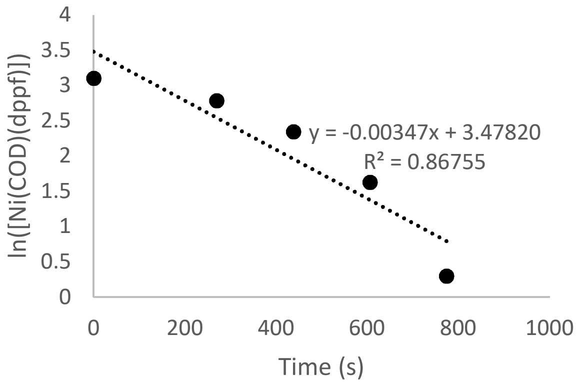

Figure S26. *Kinetic plot for Entry 6b, Table S6.*

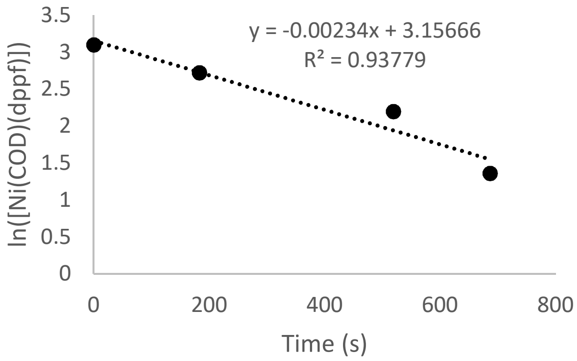

Figure S27*. Kinetic plot for Entry 7a, Table S1.*

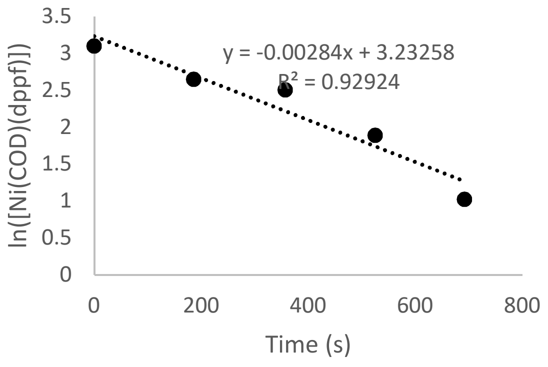

Figure S28. *Kinetic plot for Entry 7b, Table S1.*

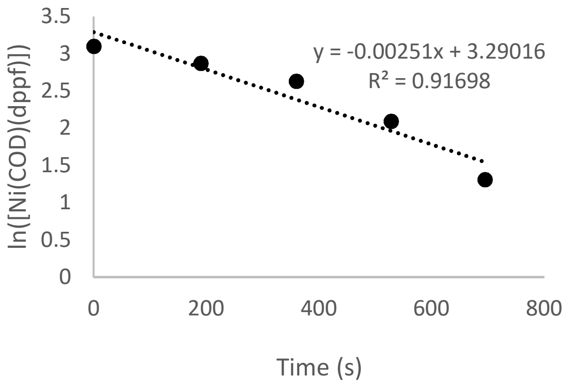

Figure S30. *Kinetic plot for Entry 8b, Table S1.*

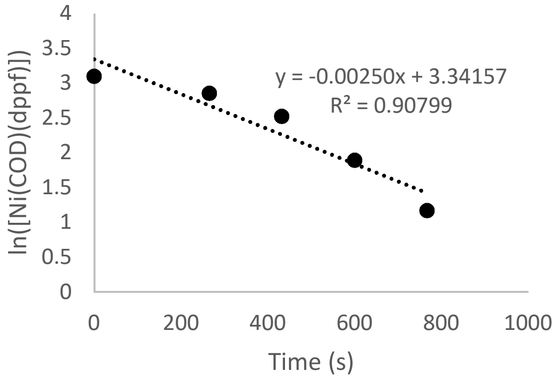

Figure S29. *Kinetic plot for Entry 8a, Table S1.*

Spectra from Reactions of 2-chloropyridine

NMR kinetic experiments of 2-chloropyridine identified signals corresponding to **3-Cl** and to other, as-yet unidentified species. A series of spectra are provided here from a representative experiment in which [Ni(COD)(dppf)] is exposed to 20 equiv. of 2-chloropyridine in benzene-d_6 at 283 K (Figure S31). Note that each spectrum is individually scaled so that the tallest peak reaches the top of the spectrum, so that the new signals can be seen clearly.

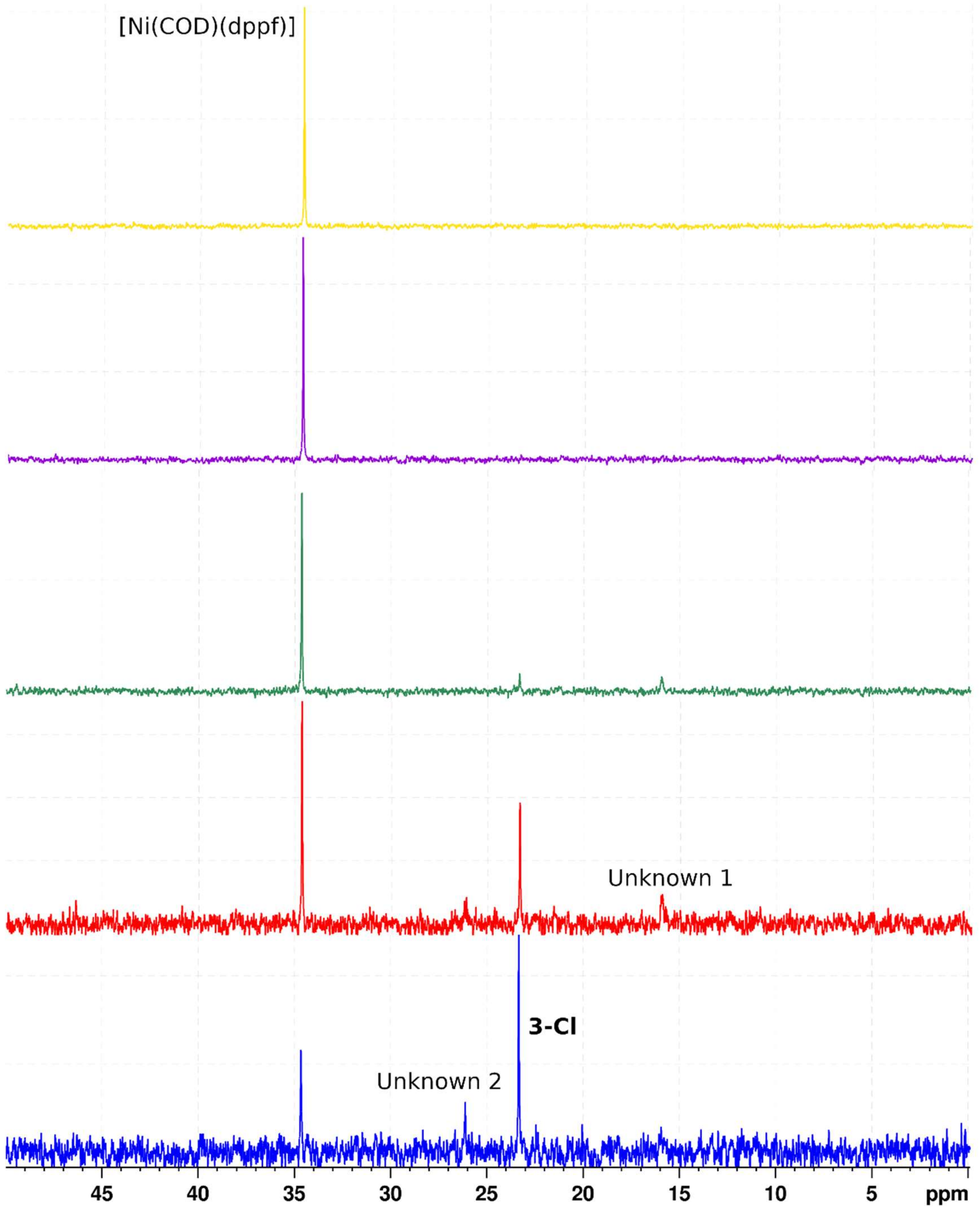

Figure S31. Stack plot from a reaction in which [Ni(COD)(dppf)] undergoes oxidative addition to 2-chloropyridine in benzene-d_6 at 283 K.

Computational Studies

General

All calculations were carried out using Gaussian16 Rev. C.01.[13] Optimisation was carried out without symmetry constraints using the B3LYP functional with Grimme D3 empirical dispersion corrections; the LANL2TZ(f) basis set was used for Ni and Fe, and the 6-31G(d) basis set was used for all other atoms. The nature of each stationary point was verified using frequency calculations. Energies were refined using single point calculations with the M06 functional and with the 6-311+G(d,p) basis set for all atoms, and with solvation modelled using the SMD model (benzene solvent). The free energies reported in this work are obtained from the sum of E at M06/6-311+G(d,p) and the correction to free energy obtained from a frequency calculation at B3LYP-D3/6-31(G)+LANL2TZ(f); a further 1.89 kcal/mol was added to each free energy to better reflect solution chemistry.[14]

Geometries

The geometries of all compounds modelled during this study can be obtained either:

(1) From the ioChem-BD[15] instance hosted at the Barcelona Supercomputing Centre, *via* the URL noted in the manuscript. This is the recommended option.

(2) From the separate supporting information file in .xyz format. This can be opened with software such as (for example) Avogadro or ChemCraft, or transferred into other files for use in software such as ORCA or Gaussian.

Energies

***Table S9**. Energies of structures considered during this work, in Hartrees.*

Structure	Code/Number	B3LYP/6-31G(d)+LANL2TZ(f)		M06/6-311+G(d,p)
		E	G_{corr}	E
Reference Complex				
[Ni(COD)(dppf)]	**2**	-2600.17101658	0.631292	-5078.28846654
Ligands				
COD		-312.040174589	0.149935	-311.861873578
dppf		-2118.70416705	0.452031	-3258.13033616
Substrates				
chlorobenzene		-691.843825029	0.061497	-691.715985299
2-chloropyridine		-707.880568901	0.049674	-707.759197681
3-chloropyridine		-707.877477736	0.049748	-707.754282032
4-chloropyridine		-707.878452059	0.049777	-707.755554751
η^2-Complexes				
[Ni(η^2-PhCl)(dppf)]	**A-PhCl**	-2979.95037771	0.538817	-5458.12537824
[Ni(η^2-2-pyCl)(dppf)]	**A_1-2pyCl**	-2995.98920669	0.526768	-5474.16923147
[Ni(η^2-2-pyCl)(dppf)]	**A_2-2pyCl**	-2995.99233536	0.526284	-5474.17273218
[Ni(η^2-3-pyCl)(dppf)]	**A-3pyCl**	-2995.99147768	0.528040	-5474.16981983
[Ni(η^2-3-pyCl)(dppf)]	**A-3pyCl_alt**[a]	-2995.98922132	0.526921	-5474.16886117
[Ni(η^2-4-pyCl)(dppf)]	**A-4pyCl**	-2995.99166854	0.526992	-5474.17193220

κ¹-Coordination via Nitrogen				
[Ni(dppf)(κ¹-*N*-2Clpy)]		-2995.983695	0.522837	-5474.161926
[Ni(dppf)(κ¹-*N*-3Clpy)]		-2995.980646	0.521686	-5474.156171
[Ni(dppf)(κ¹-*N*-4Clpy)]		-2995.981315	0.521478	-5474.157062
κ¹-Coordination via Chlorine				
[Ni(dppf)(κ¹-*Cl*-ClPh)]		-2979.928308	0.534113	-5458.103721
[Ni(dppf)(κ¹-*Cl*-2Clpy)]		-2995.963852	0.523435	-5474.145441
[Ni(dppf)(κ¹-*Cl*-3Clpy)]		-2995.962100	0.522169	-5474.141325
[Ni(dppf)(κ¹-*Cl*-4Clpy)]		-2995.961679	0.524097	-5474.141541
Oxidative Addition TS				
[Ni(dppf)] + PhCl	**TS-A-B-PhCl**	-2979.93338639	0.537619	-5458.10279803
[Ni(dppf)] + 2-pyCl *via* **A₁**	**TS-A₁-B-2py**	-2995.97520184	0.526107	-5474.14984138
[Ni(dppf)] + 2-pyCl *via* **A₂**	**TS-A₂-B'-2py**	-2995.98954531	0.523922	-5474.16886068
[Ni(dppf)] + 3-pyCl *via* **A**	**TS-A-B-3py**	-2995.96945084	0.525309	-5474.14319087
[Ni(dppf)] + 3-pyCl *via* **A_alt**	**TS-A_alt-B-3py**[a]	-2995.96843923	0.525354	-5474.14237200
[Ni(dppf)] + 4-pyCl	**TS-A-B-4py**	-2995.97248564	0.526161	-5474.14760069
Square Planar Products				
[NiCl(Ph)(dppf)]	**B-PhCl**	-2979.98524187	0.539267	-5458.15723953
[NiCl(2-py)(dppf)]	**B-2py**	-2996.02751813	0.526792	-5474.20503826
[NiCl(3-py)(dppf)]	**B-3py**	-2996.02302292	0.527261	-5474.20149282
[NiCl(4-py)(dppf)]	**B-4py**	-2996.02521988	0.527637	-5474.20340187
Square-based Pyramid				
[NiCl(κ²-2py)(dppf)]	**B'-2py**	-2996.01487493	0.526517	-5474.19243809
Rearrangement TS				
[NiCl(κ²-2py)(dppf)] to [NiCl(2-py)(dppf)]	**TS-B-B'-2py**	-2996.02864374	0.526908	-5474.18838615
*Pathway to Form **3-Cl***				
[NiCl(κ²-2py)(κ¹-dppf)] isomer 1	**C**	-2996.011498	0.520779	-5474.191594
[NiCl(κ²-2py)(κ¹-dppf)] isomer 2[a]		-2996.008232	0.521704	-5474.188002
[{NiCl(κ¹-dppf)}(μ-2py){NiCl(2-py)(κ²-dppf)]	**D**	-5992.092636	1.083347	-10948.41883
[{NiCl(κ¹-dppf)}(μ-2py){NiCl(2-py)(κ¹-dppf)]	**E**	-5992.066875	1.075211	-10948.39326
[NiCl(μ-2-py)(κ¹-dppf)]₂	**F**	-5992.073163	1.080166	-10948.39441
[NiCl(μ-2-py)(κ¹-dppf)NiCl(μ-2py)]	**G**	-3873.320542	0.600045	-7690.241448
[{NiCl(μ-2-py)}₂{μ-dppf}]	**3-Cl**	-3873.387550	0.604729	-7690.303653
Alternative Ligands				
[Ni(COD)₂]		-793.478664160	0.325230	-2132.00073282

PPh_3		-1036.31282784	0.227937	-1035.91975486
PCy_3		-1047.21883064	0.440186	-1046.79750201
dppe		-1687.95082972	0.365523	-1687.37344146
XantPhos		-2263.41306126	0.526483	-2262.54586272
[NiCl(2-py)(PPh_3)$_2$]		-2949.98021849	0.554340	-4287.93239181
[NiCl(2-py)(PCy_3)$_2$]		-2971.79224755	0.979887	-4309.68283127
[NiCl(2-py)(dppe)]		-2565.27682918	0.436586	-3903.41024368
[NiCl(2-py)(XantPhos)]		-3140.73625018	0.601234	-4478.61556446
PPh_3 dimer		-3827.33477695	0.632096	-6504.02511167
PCy_3 dimer		-3849.13427878	1.059894	-6525.76634731
dppe dimer		-3442.61865398	0.515713	-6119.53361709
XantPhos dimer		-4018.08563110	0.681851	-6694.70757820

[a] This structure is higher in energy than the alternative isomer, but is included for completeness.

QTAIM Analysis

Figure S32. **TS-A$_2$-B’** (2-chloropyridine)

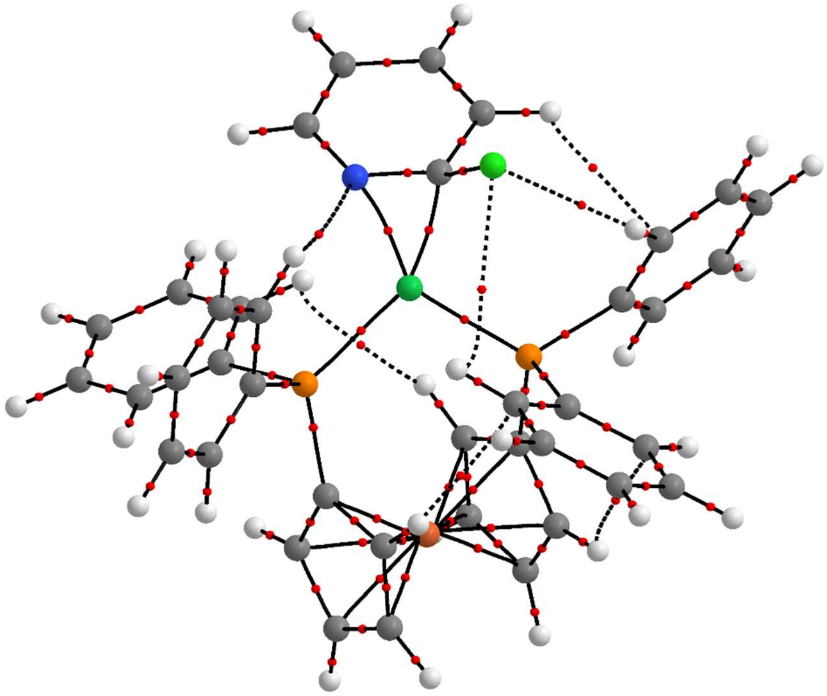

Figure S33. TS-A$_1$-B (2-chloropyridine)

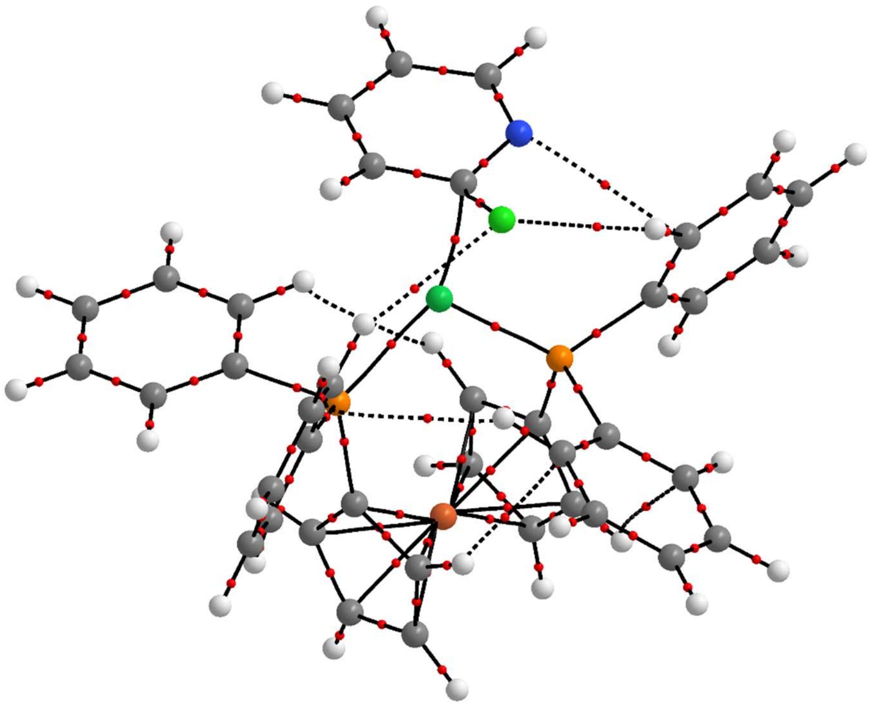

Figure S34. TS-A-B (chlorobenzene)

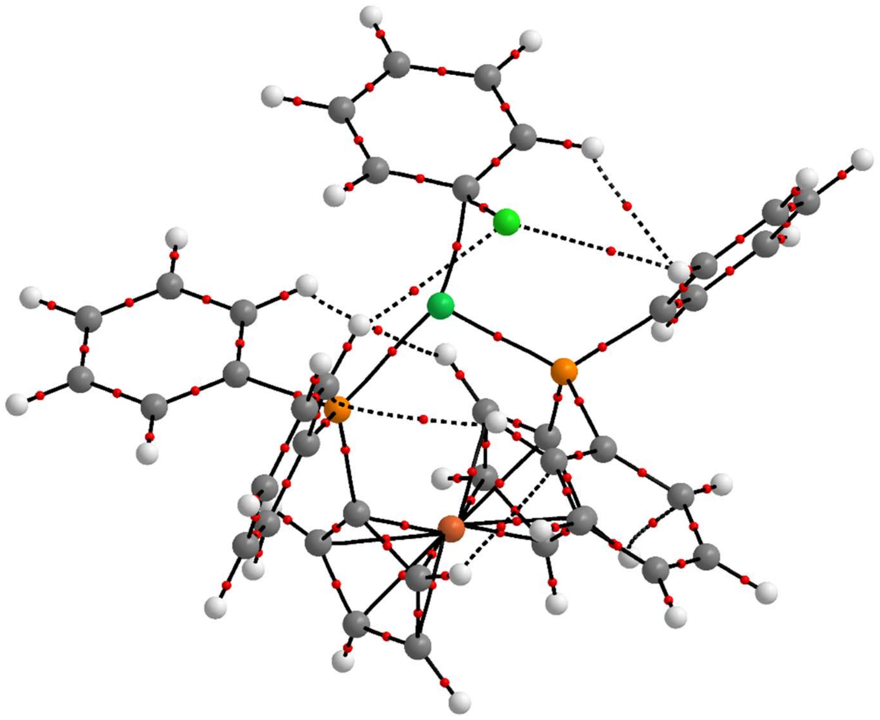

Figure S35. 3-Cl (from 2-chloropyridine)

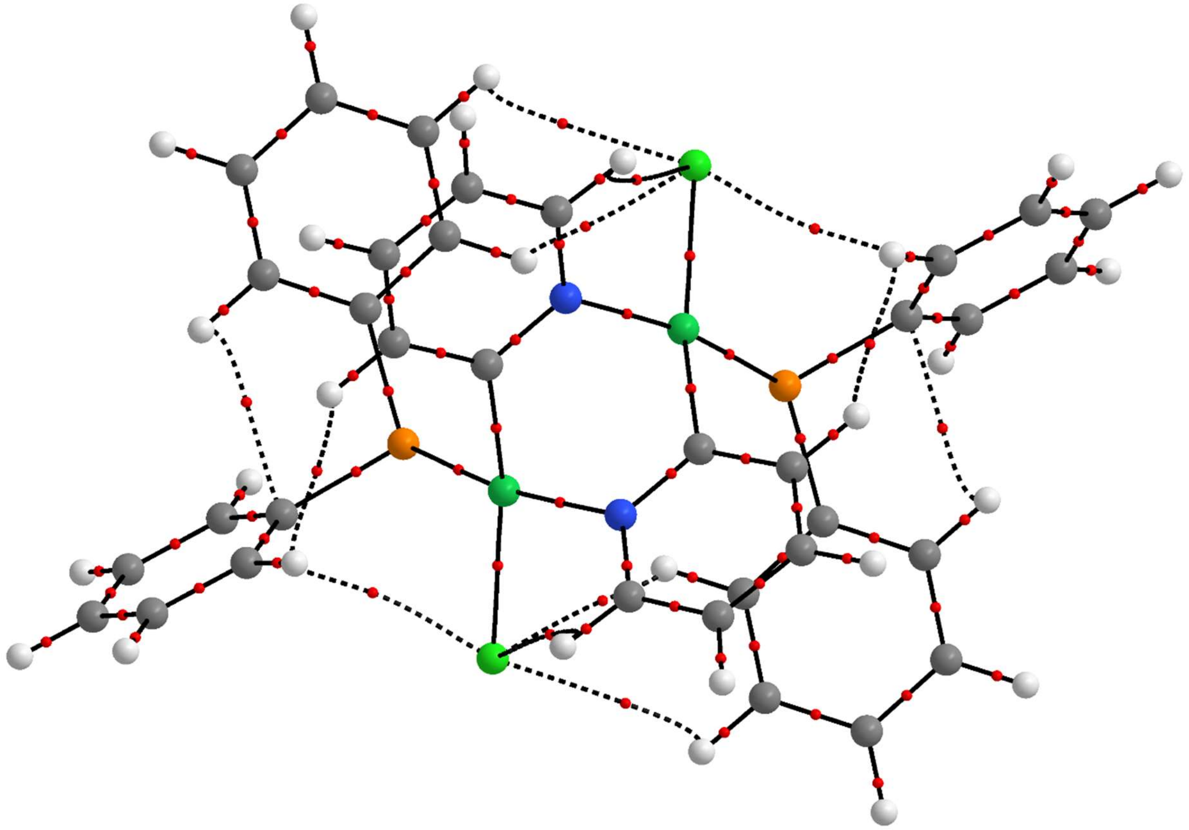

NB: The ferrocene fragment is omitted for clarity.

References

1. E. A. Standley, S. J. Smith, P. Müller and T. F. Jamison, *Organometallics*, 2014, **33**, 2012-2018.
2. G. Yin, I. Kalvet, U. Englert and F. Schoenebeck, *J. Am. Chem. Soc.*, 2015, **137**, 4164-4172.
3. J. Yang, S. Liu, J.-F. Zheng and J. Zhou, *Eur. J. Org. Chem.*, 2012, **2012**, 6248-6259.
4. D. E. Frantz, D. G. Weaver, J. P. Carey, M. H. Kress and U. H. Dolling, *Org. Lett.*, 2002, **4**, 4717-4718.
5. X.-H. Xu, X. Wang, G.-k. Liu, E. Tokunaga and N. Shibata, *Org. Lett.*, 2012, **14**, 2544-2547.
6. CrysalisPro (Rigaku Oxford Diffraction, 2019)
7. G. Sheldrick, *Acta Crystallographica Section C*, 2015, **71**, 3-8.
8. L. J. Farrugia, *J. Appl. Cryst.*, 2012, **45**, 849-854.
9. S. Bajo, G. Laidlaw, A. R. Kennedy, S. Sproules and D. J. Nelson, *Organometallics*, 2017, **36**, 1662-1672.
10. A. K. Cooper, D. K. Leonard, S. Bajo, P. M. Burton and D. J. Nelson, *Chem. Sci.*, 2020, **11**, 1905-1911.
11. M. E. Greaves, T. O. Ronson, G. C. Lloyd-Jones, F. Maseras, S. Sproules and D. J. Nelson, *ACS Catal.*, 2020, **10**, 10717-10725.
12. M. E. Greaves, T. O. Ronson, F. Maseras and D. J. Nelson, *Organometallics*, 2021, **40**, 1997-2007.
13. M. J. Frisch, G. W. Trucks, H. B. Schlegel, G. E. Scuseria, M. A. Robb, J. R. Cheeseman, G. Scalmani, V. Barone, G. A. Petersson, H. Nakatsuji, X. Li, M. Caricato, A. V. Marenich, J. Bloino, B. G. Janesko, R. Gomperts, B. Mennucci, H. P. Hratchian, J. V. Ortiz, A. F. Izmaylov, J. L. Sonnenberg, Williams, F. Ding, F. Lipparini, F. Egidi, J. Goings, B. Peng, A. Petrone, T. Henderson, D. Ranasinghe, V. G. Zakrzewski, J. Gao, N. Rega, G. Zheng, W. Liang, M. Hada, M. Ehara, K. Toyota, R. Fukuda, J. Hasegawa, M. Ishida, T. Nakajima, Y. Honda, O. Kitao, H. Nakai, T. Vreven, K. Throssell, J. A. Montgomery Jr., J. E. Peralta, F. Ogliaro, M. J. Bearpark, J. J. Heyd, E. N. Brothers, K. N. Kudin, V. N. Staroverov, T. A. Keith, R. Kobayashi, J. Normand, K. Raghavachari, A. P. Rendell, J. C. Burant, S. S. Iyengar, J. Tomasi, M. Cossi, J. M. Millam, M. Klene, C. Adamo, R. Cammi, J. W. Ochterski, R. L. Martin, K. Morokuma, O. Farkas, J. B. Foresman and D. J. Fox, *Journal*, 2016.
14. J. N. Harvey, F. Himo, F. Maseras and L. Perrin, *ACS Catal.*, 2019, **9**, 6803-6813.
15. M. Álvarez-Moreno, C. de Graaf, N. López, F. Maseras, J. M. Poblet and C. Bo, *J. Chem. Inf. Model.*, 2015, **55**, 95-103.
